# Challenging dynamic cerebral autoregulation across the physiological CO_2_ spectrum: Influence of biological sex and cardiac cycle

**DOI:** 10.1113/EP092245

**Published:** 2024-11-18

**Authors:** Nathan E. Johnson, Joel S. Burma, Matthew G. Neill, Joshua J. Burkart, Elizabeth K. S. Fletcher, Jonathan D. Smirl

**Affiliations:** ^1^ Cerebrovascular Concussion Lab, Faculty of Kinesiology University of Calgary Calgary Alberta Canada; ^2^ Sport Injury Prevention Research Centre, Faculty of Kinesiology University of Calgary Calgary Alberta Canada; ^3^ Human Performance Laboratory, Faculty of Kinesiology University of Calgary Calgary Alberta Canada; ^4^ Libin Cardiovascular Institute of Alberta University of Calgary Calgary Alberta Canada; ^5^ Alberta Children's Hospital Research Institute University of Calgary Calgary Alberta Canada; ^6^ Hotchkiss Brain Institute University of Calgary Calgary Alberta Canada; ^7^ Integrated Concussion Research Program University of Calgary Calgary Alberta Canada

**Keywords:** dynamic end‐tidal forcing, eucapnia, hypercapnia, hypocapnia, sex differences, squat–stand manoeuvres

## Abstract

This study applied alterations in partial pressure of end‐tidal carbon dioxide ($P_{\mathrm{ETC}O_{2}}$) to challenge dynamic cerebral autoregulation (dCA) responses across the cardiac cycle in both biological sexes. A total of 20 participants (10 females and 10 males; aged 19–34 years) performed 4‐min bouts of repeated squat–stand manoeuvres (SSMs) at 0.05 and 0.10 Hz (randomized orders) with $P_{\mathrm{ETC}O_{2}}$ clamped at ∼40 mmHg. The protocol was repeated for hypercapnic (∼55 mmHg) and hypocapnic (∼20 mmHg) conditions. Middle cerebral artery (MCA) and posterior cerebral artery (PCA) were insonated via transcranial Doppler ultrasound. Dynamic end‐tidal forcing clamped $P_{\mathrm{ETC}O_{2}}$, and finger photoplethysmography quantified beat‐to‐beat changes in blood pressure. Linear regressions were performed for transfer function analysis metrics including power spectrum densities, coherence, phase, gain and normalized gain (nGain) with adjustment for sex. During hypercapnic conditions, phase metrics were reduced from eucapnic levels (all *P <* 0.009), while phase increased during the hypocapnic stage during both 0.05 and 0.10 Hz SSMs (all *P <* 0.037). Sex differences were present with females displaying greater gain and nGain systole metrics during 0.10 Hz SSMs (all *P <* 0.041). Across $P_{\mathrm{ETC}O_{2}}$ stages, females displayed reduced buffering against systolic aspects of the cardiac cycle and augmented gain. Sex‐related variances in dCA could explain sex differences in the occurrence of clinical conditions such as orthostatic intolerance and stroke, though the effect of fluctuating sex hormones and contraceptive use on dCA metrics is not yet understood.

## Abstract (highlights)

- **What is the central question of this study?**
  What is the influence partial pressure of end tidal carbon dioxide ($P_{\mathrm{ETC}O_{2}}$) on dynamic cerebral autoregulation (dCA) across all aspects of the cardiac cycle during hypocapnia, eucapnia and hypercapnia, and are biological sex differences observed in the responses?
- **What is the main finding and its importance?**
  There was delayed dCA response in hypercapnia and enhanced dCA during hypocapnia. Biological sex differences were most evident during 0.10 Hz squat–stand manoeuvres. Middle cerebral artery systolic gain and nGain were lower in males compared to females.

## 1 INTRODUCTION

While occupying just 2% of total body weight, the human brain is a highly complex organ with a substantial metabolic demand (Willie et al., 2014). To maintain optimal functioning, the brain utilizes ∼20% of both cardiac output and total oxygen consumed at rest (Rink & Khanna, 2011; Williams & Leggett, 1989). Given its limited storage capacity for essential substances, the brain depends on several intricate regulatory mechanisms to maintain cerebral perfusion (Willie et al., 2014). One of the key regulatory mechanisms is dynamic cerebral autoregulation (dCA). dCA describes the brain's ability to appropriately regulate cerebral blood flow (CBF) and perfusion pressure during temporary changes in systemic blood pressure (Brassard et al., 2021) to mitigate hypo‐ and hyper‐perfusion (Brassard et al., 2023; Numan et al., 2014; Willie et al., 2014). Furthermore, there is evidence of a greater dCA buffering associated with the systolic aspect of the cardiac cycle as compared to diastole (Burma et al., 2020, 2020; Newel et al., 2022; Smirl et al., 2018). Dysfunction in this regulatory system is associated with various clinical conditions, including orthostatic intolerance, hypotension and exercise intolerance (Claassen et al., 2021).

The brain is also highly sensitive to fluctuations in circulating arterial CO_2_ levels, with hypercapnia and hypocapnia resulting in substantial vasodilatation and vasoconstriction responses, respectively (Ainslie & Hoiland, 2014; Fierstra et al., 2013; Kety & Schmidt, 1948). This second regulatory mechanism, known as cerebrovascular reactivity (CVR), describes the cerebrovascular response to vasoactive stimuli (Fierstra et al., 2013). While initial CVR studies employed breath hold or rebreathing techniques (Fierstra et al., 2013) to manipulate circulating arterial CO_2_, studies have demonstrated the effectiveness of dynamic end‐tidal forcing (DEF) in controlling and clamping end‐tidal gases using computer‐based algorithms (O'Connor et al., 2016; Tymko et al., 2015). Through the use of feed‐forward algorithms, DEF can precisely manipulate the end‐tidal pressure of CO_2_ ($P_{\mathrm{ETC}O_{2}}$) and the end‐tidal pressure of oxygen ($P_{\mathrm{ET}O_{2}}$) by adjusting the fraction of inspired CO_2_ and oxygen on a breath‐by‐breath basis to maintain these at the desired levels (Tymko et al., 2015). This approach allows for more temporally and volumetrically precise titration of CO_2_, allowing for higher resolution investigation of CVR and hypo‐/hypercapnic dCA.

A previous study by Birch et al. (1995) examined repeated squat–stand manoeuvres (SSMs) during eucapnic, hypercapnic (induced via 5% CO_2_ gas inhalation) and hypocapnic (induced via hyperventilation) conditions and noted reductions in phase metrics during hypercapnia and increases in phase during hypocapnia in the middle cerebral artery (MCA). Building upon this, Smirl, Tzeng et al. (2014) observed both indomethacin and hypocapnia (induced via hyperventilation) produced significant vasoconstriction, resulting in more tightly controlled dCA responses in both the MCA and the posterior cerebral artery (PCA). However, these studies only reported means without including specific measures across the cardiac cycle. How the diastolic and systolic aspects of dCA across the cardiac cycle are affected by DEF alterations to CO_2_ is currently unknown but may shed light on the mechanistic differences between sexes and the mechanisms underlying cerebrovascular dysfunction.

Furthermore, there is evidence of biological sex differences in dCA metrics during eucapnic breathing challenges (Deegan et al., 2009; Favre & Serrador, 2019). Additionally, sex differences have emerged when observing different aspects of the cardiac cycle, with females displaying reduced systolic dCA regulation during SSMs performed with additional resistance (Newel et al., 2022). However, there is a paucity of research exploring the influence of hypercapnia or hypocapnia across the cardiac cycle during SSMs and the subsequent effects of CVR on dCA across biological sexes.

Therefore, the purpose of the current investigation was two‐fold. First, this study sought to investigate the impact of CVR (hypocapnia, eucapnia and hypercapnia) across the physiological spectrum of $P_{\mathrm{ETC}O_{2}}$ levels via DEF on dCA across the cardiac cycle during SSMs performed at 0.05 and 0.10 Hz. Additionally, the study aimed to understand potential biological sex differences in the dCA to various CVR challenges. It was hypothesized during the hypercapnic breathing challenges that both males and females would display reduced phase and increased gain dCA relative to eucapnia levels. However, the opposite was hypothesized for the hypocapnic challenges, with both males and females expected to demonstrate greater dCA control evidenced by increased phase and reduced gain dCA metrics compared to the eucapnic stage. During eucapnic trials, females are expected to display less buffering ability within the systolic aspect of the cardiac cycle (i.e., greater gain) (Newel et al., 2022) and these biological sex differences were hypothesized to remain consistent under hypercapnic and hypocapnic conditions.

## 2 METHODS

### 2.1 Ethical approval

The University of Calgary Conjoint Health Research Ethics Board (REB20‐1662 and REB 20–2112) provided the ethical approval for the current investigation. Prior to beginning data collection, investigators thoroughly explained the protocol and the equipment used for collecting. Furthermore, each participant provided written informed consent before beginning data collection. Finally, all procedures were followed according to the institutional guidelines and those put forth by the *Declaration of Helsinki* (World Medical Association, 2013), aside from study registration within a database.

### 2.2 Participants and study design

The current investigation recruited 20 healthy adults (10 females and 10 males) between the ages of 19 and 34 for one laboratory visit. While participants were asked to report both self‐reported biological sex and the social construct of gender, all participants of this investigation reported as *cis*‐gendered, and therefore all data will be discussed in reference to biological sex. The female mean age was 21.6 ± 1.1 years, with an average body mass index (BMI) of 25.4 ± 4.2 kg/m^2^. The male mean age was 25.1 ± 4.3 years, with an average BMI of 24.1 ± 2.0 kg/m^2^. Participants were included if they were free from cardiorespiratory, musculoskeletal, neurological and/or cerebrovascular conditions and had not suffered a concussion within the last 6 months. On the day of the study, participants were required to abstain from caffeine, alcohol, smoking and vaping for a minimum of 8 h before beginning data collection (Ainslie et al., 2007, 2008; Smirl et al., 2015; Smirl, Haykowsky et al., 2014) as well as refrain from exercise 6 h prior to testing (Burma, Copeland, Macaulay, Khatra, Wright et al., 2020).

### 2.3 Experimental protocols

The data collection visit occurred at the Cerebrovascular Concussion Laboratory, University of Calgary, located 1111 m above sea level. Participants’ sex, age, height and weight were recorded after obtaining written consent. Participants then completed a comprehensive repeated‐measures protocol, consisting of a neurovascular coupling task, the *Where's Waldo* search paradigm (Burma, Wassmuth et al., 2021; Smirl et al., 2016). Those data will be published elsewhere. Subsequently, participants performed SSMs at frequencies of 0.05 and 0.10 Hz) (Panerai et al., 2023) while end‐tidal gases were clamped using DEF at hypocapnic (∼25 mmHg), eucapnic (∼40 mmHg) and hypercapnic (∼55 mmHg) levels. During testing, the barometric pressure was 666.4 ± 4.4 mmHg, the humidity was 16.8 ± 6.9%, and the room temperature was 22.0 ± 0.7°C.

The testing session began with the eucapnic breathing challenge, where $P_{\mathrm{ETC}O_{2}}$ was clamped at 40 mmHg. Participants performed one trial of 4‐min sets of SSMs at each 0.05 and 0.10 Hz frequency in a randomized order, which has been proven to provide valid and reliable transfer function analysis (TFA) metrics (Burma, Miutz et al., 2021). The protocol was repeated at hypercapnic levels, clamping $P_{\mathrm{ETC}O_{2}}$ at 55 mmHg, followed by hypocapnic levels with $P_{\mathrm{ETC}O_{2}}$ clamped at 25 mmHg. The clamped $P_{\mathrm{ETC}O_{2}}$ levels were selected based on previous literature that demonstrated 25–30 mmHg to be a safe benchmark for measuring hypocapnia, and 55 mmHg to be sufficient for inducing hypercapnia at low altitudes (Tymko et al., 2015). Moreover, a brief 5‐min ‘washout’ period between hyper‐ and hypo‐capnic stages was provided allowing $P_{\mathrm{ETC}O_{2}}$ to return to baseline (Burma, Macaulay, Copeland, Khatra, Bouliane et al., 2020). The sequence of conditions (eucapnia, hypercapnia and hypocapnia) was selected based on previous findings suggesting that vasoconstriction responses remain unaffected following moderate to high‐intensity exercise, which induces vasodilatation similar to hypercapnia (Burma, Macaulay, Copeland, Khatra, Bouliane et al., 2020). The total testing duration was approximately 1.5 h and was completed in a single visit between December 2023 and February 2024.

The selection of the aforementioned 0.05 Hz SSMs was based on their positioning within the very‐low‐frequency range of dCA (0.02–0.07 Hz) (Claassen et al., 2016; Panerai et al., 2023; Zhang et al., 1998). This range enables the assessment of dCA impact on various responses including endothelial, neurogenic, metabolic and myogenic responses (Hamner et al., 2010; Hamner & Tan, 2014). Furthermore, 0.10 Hz SSMs were selected as this frequency falls within the low‐frequency range (0.07–0.20 Hz) (Claassen et al., 2016; Panerai et al., 2023; Zhang et al., 1998). This frequency range allows examination of the influence of sympathetic innervation on dCA (Hamner et al., 2010; Hamner & Tan, 2014). During data collection, a metronome set to either 6 beats/min (0.05 Hz) or 12 beats/min (0.10 Hz) aided participants in performing SSMs at the appropriate frequency and completing their transitions in ∼1–2 s (Newel et al., 2022).

### 2.4 Instrumentation

During the data collection set‐up, the participants’ MCA and posterior PCA were insonated unilaterally using transcranial Doppler ultrasound (TCD) (DWL USA, Inc., San Juan Capistrano, CA, USA) to monitor cerebral blood velocity (CBv: index of CBF) (Skow et al., 2022). The two 2 MHz ultrasound probes were positioned at the transtemporal window to insonate the P1 and M2 segments of the left PCA and right MCA, respectively. The vessels were insonated by highly trained sonographers who utilized carotid compressions, and simple visual tasks were used to verify that the correct vessels were insonated. After, the TCD headframe was used to lock the probes in place and maintain positioning throughout testing (DWL USA). A finger photoplethysmography device with a height correct unit (Finometer NOVA; Finapres Medical Systems, Amsterdam, The Netherlands) was used to record blood pressure on a beat‐to‐beat basis and pulsatile waveforms corrected for differences in hand height relative to the heart. During the protocol, a portable DEF system was utilized to regulate $P_{\mathrm{ETC}O_{2}}$ and $P_{\mathrm{ET}O_{2}}$ on a breath‐by‐breath basis (Tymko et al., 2015). This system utilizes independent solenoid valves for nitrogen, oxygen, and CO_2_, to ensure precise control over $P_{\mathrm{ET}O_{2}}$ and $P_{\mathrm{ETC}O_{2}}$ metrics through feedback and feedforward mechanisms. Further, an end‐tidal steady state was achieved when $P_{\mathrm{ETC}O_{2}}$ was within 1 mmHg of the target stage for three consecutive breaths, which in turn will maintain consistent arterial gas levels. The DEF system offers advantages such as the ability to maintain end‐tidal CO_2_ levels accurately at desired values, and enhancing control through adjusting volume injected into the inspiratory reserve through feedback and feedforward mechanisms (O'Connor et al., 2016; Tymko et al., 2015). Participants wore a nose clip while they breathed into a mouthpiece attached to a sampling line that ran the gas analyser (ML206; ADInstruments, Colorado Springs, CO, USA). Finally, all data were simultaneously sampled at 1000 Hz by an analog‐to‐digital conversion (PowerLab 16/30 ML880; ADInstruments). Commercially available software was used to store the data for analyses (LabChart Pro Version 8, ADInstruments).

### 2.5 Data processing

Consistent with previous studies, blood pressure and CBv values were calculated throughout the cardiac cycle (Burma, Copeland, Macaulay, Khatra, Wright et al., 2020; Newel et al., 2022; Panerai et al., 2023). The maximum and minimum points from each pulsative waveform were used to obtain systolic and diastolic values, respectively. The average of all data points calculated across the waveform was used to obtain mean values. The data, sampled at 1000 Hz using Powerlab 16/30 ML880 software (ADInstruments), underwent visual inspection by the investigators to manually correct artifacts by interpolating systolic peaks, which was required for less than 0.1% of all traces. Further, heart rate was derived from the R‐R interval, while $P_{\mathrm{ETC}O_{2}}$ calculations used breath‐to‐breath peak values of partial pressure of carbon dioxide.

The data processing stage was performed while complying with the standards put forward by the Cerebral Autoregulation Research Network (CARNet) white papers (Claassen et al., 2016; Panerai et al., 2023). LabChart (ADInstumnets) quantified the physiological outputs during the SSMs, including mean arterial blood pressure, CBv, $P_{\mathrm{ETC}O_{2}}$ and so forth. Additionally, commercially available software (Ensemble‐R V1.0.42, R&D Canvas, Wellington, New Zealand) was employed to derive CA power spectrum density (PSD) and TFA metrics.

### 2.6 TFA and power spectrum densities

Welch's smoothing method was employed to obtain spectral estimates. This included spline interpolating and resampling beat‐to‐beat BP and CBv waveforms at 4 Hz (Claassen et al., 2016; Panerai et al., 2023). Based on previous findings, a 240‐s recording was utilized to obtain TFA estimates from the SSMs, as this time frame has been demonstrated to be sufficient for obtaining reliable and valid metrics (Burma, Miutz et al., 2021). After, the data underwent detrending and Hanning window application (four windows, 50% overlap, 80 s per window). Cross‐spectrums, as previously described (Claassen et al., 2016; Panerai et al., 2023; Zhang et al., 1998), were computed between BP and CBv at different phases of the cardiac cycle, such as diastolic arterial blood pressure and diastolic CBv. The data of each cardiac cycle component were then normalized by their respective autospectrum (Smirl et al., 2018; Wright et al., 2018). Additionally, cerebral autoregulatory TFA estimates were subsequently calculated at specific frequency‐point‐estimates (0.05 and 0.10 Hz) for diastolic, mean and systolic aspects of the cardiac cycle (Burma, Copeland, Macaulay, Khatra, Wright et al., 2020; Panerai et al., 2023).

The primary outcome measures of interest were the Fourier transformation PSD, and TFA‐derived metrics of coherence, phase, gain and normalized gain. Power spectrum density was used to quantify signal strength in the frequency domain (Zhang et al., 1998). Using an arbitrary unit scale from 0 to 1, coherence was used to describe the linear input–output relationship of the cerebral pressure–flow relationship, which ranges from no linear relationship to a complete linear relationship, respectively (Zhang et al., 1998). Furthermore, phase (radians) indicates temporal alignment, while absolute gain (cm/s/mmHg) measures amplitude modulation in the cerebral pressure–flow relationship (Zhang et al., 1998). Recently, previous research has noted the ability of normalized gain (%/mmHg) to display increased reliability, and therefore to complement this work we included it in the analysis (Burma et al., 2020; Smirl et al., 2015). The aforementioned TFA computation method used spectral smoothing, 50% overlap and five windows; the coherence's critical value aligned with CARNet White Paper recommendations (0.46), and was set at an alpha value of 0.01 (Panerai et al., 2023). No phase wraparound was observed in any data samples.

### 2.7 Sample size calculation

G*Power (Version 3.1.9.6) was used to determine an a priori sample size using a multiple linear regression model. The primary interest of the current investigation was to determine the influence of $P_{\mathrm{ETC}O_{2}}$ on the dCA capabilities during different frequencies of SSMs. A previous report by Smirl, Tzeng, and colleagues (2014) noted that both indomethacin and hypocapnia produced large effect size changes in TFA metrics of interest (i.e., phase and gain). Therefore, using a large Cohen's *f* ^2^ effect size of 0.35, an α of 0.05, a subsequent power of 0.80, a one‐tailed test (due to the hypotheses), and two predictor variables (condition and sex), a sample size of 18 was required to perform a linear model. However, as linear mixed‐effects models (LME) increase power (Bagiella et al., 2000), this sample would theoretically be sufficient for LME modelling as well. Notably, there is no consistent agreement on the a priori sample size for LME models (Matuschek et al., 2017).

### 2.8 Statistical analysis

All statistical analyses were conducted using R‐Studio (Version 2024.04.2+764). LME models with participants included as random effects were performed for all TFA outcome metrics. The fixed effect predictor variables included $P_{\mathrm{ETC}O_{2}}$ stages (eucapnia [reference], hypercapnia, hypocapnia) and sex (female [reference], male) across the cardiac cycle. Additionally, each LME model was compared against a null model with no fixed effects using a likelihood ratio (LR) test, which provided the *P*‐values for the LR test (Table 2). Data are presented as means ± 95% confidence intervals (95% CI). α‐Level was set a priori at 0.05.

## 3 RESULTS

Female mean age was 21.6 ± 1.1 years, with an average BMI of 25.4 ± 4.2 kg/m^2^. Male mean age was 25.1 ± 4.3 years, with an average BMI of 24.1 ± 2.0 kg/m^2^.

### 3.1 Physiological data

**FIGURE 1:**
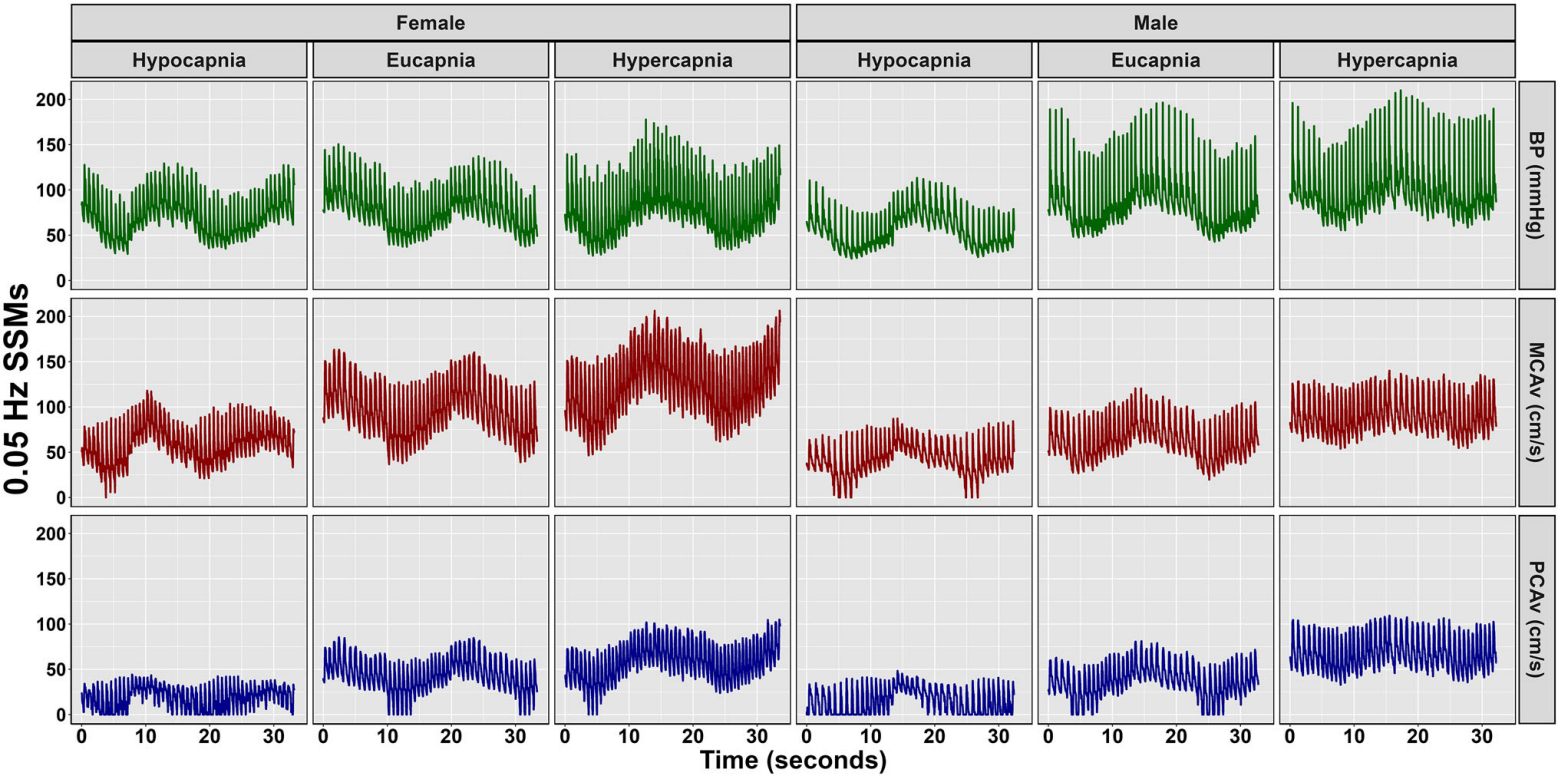
Representative blood pressure (BP), middle cerebral artery velocity (MCAv), and posterior cerebral artery velocity (PCAv) data from one female and one male participant during 0.05 Hz squat‐stand manoeuvres.

**FIGURE 2:**
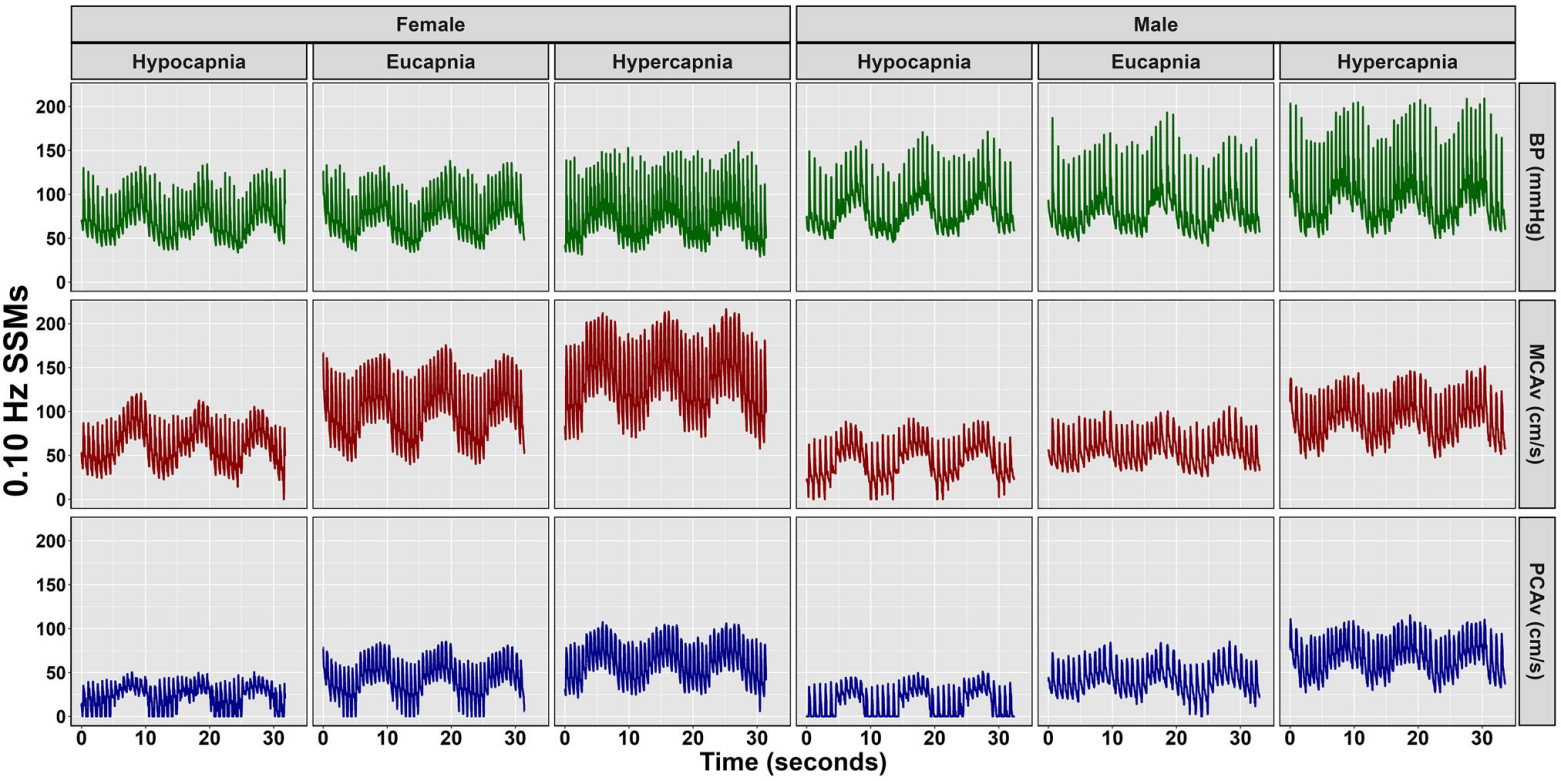
Representative blood pressure (BP), middle cerebral artery velocity (MCAv), and posterior cerebral artery velocity (PCAv) data from one female and one male participant during 0.10 Hz squat–stand manoeuvres.

Mean and 95% confidence interval biological sex‐stratified cerebrovascular, respiratory and cardiovascular metrics are displayed in Table 1. Table 2 presents outputs from LME models with participants as a random effect, including β‐coefficients, 95% CI, *P*‐values and LR *P*‐values for comparisons between hypercapnic and hypocapnic conditions against eucapnia (reference) and males against females (reference) across all stages. Absolute TFA estimates are displayed in Table 3. Representative data for BP, MCAv and PCAv taken from one female and one male participant during 0.05 and 0.10 Hz are depicted in Figures 1 and 2, respectively.

**TABLE 1:** Biological sex‐stratified cerebrovascular, respiratory and cardiovascular metrics from 20 participants (10 females and 10 males) during a repeated squat–stand manoeuvres protocol with two randomized frequencies (0.05 and 0.10 Hz) under three conditions (hypocapnia, eucapnia, hypercapnia).

Variable	Frequency	Sex	Hypocapnia	Eucapnia	Hypercapnia	Group	Sex
$P_{\mathrm{ETC}O_{2}}$ (mmHg)	0.05 Hz	Female	**28.3 (27.4, 29.2)**	38.8 (37.6, 40.0)	**52.4 (50.4, 54.3)**	***F*** = **670.7; *P <* 0.001;** $\eta_{G}^{2}$ = **0.96**	*F *= 0.1; *P *= 0.819; $\eta_{G}^{2}$ = 0.00
		Male	**26.9 (25.5, 28.3)**	39.6 (37.9, 41.3)	**52.6 (50.8, 54.4)**		
	0.10 Hz	Female	**28.2 (27.1, 29.2)**	39.6 (38.2, 41.0)	**52.4 (51.4, 53.5)**	***F*** = **618.7; *P <* 0.001;** $\eta_{G}^{2}$ = **0.96**	*F *= 0.2; *P *= 0.671; $\eta_{G}^{2}$ = 0.00
		Male	**28.1 (26.9, 29.3)**	40.1 (38.2, 42.0)	**52.7 (50.2, 55.2)**		
Respiration rate (breaths/min)	0.05 Hz	Female	16.9 (12.5, 21.4)	19.6 (16.0, 23.1)	22.3 (16.5, 28.1)	*F *= 2.9; *P *= 0.066; $\eta_{G}^{2}$ = 0.09	*F *= 0.1; *P *= 0.958; $\eta_{G}^{2}$ = 0.00
		Male	17.4 (11.9, 22.8)	19.5 (14.1, 24.9)	21.7 (19.3, 24.1)		
	0.10 Hz	Female	18.0 (12.9, 23.0)	18.4 (16.4, 20.4)	**21.8 (16.2, 27.3)**	***F*** = **5.4; *P *= 0.007;** $\eta_{G}^{2}$ = **0.16**	*F *= 0.1; *P *= 0.737; $\eta_{G}^{2}$ = 0.00
		Male	17.1 (12.5, 21.8)	18.2 (16.1, 20.4)	**24.3 (20.6, 27.9)**		
Diastolic MCAv ((cm/s)^2^/Hz)	0.05 Hz	Female	**42.6 (32.9, 52.2)**	60.2 (49.9, 70.5)	**86.0 (63.7, 108)**	***F*** = **30.2; *P <* 0.001;** $\eta_{G}^{2}$ = **0.52**	***F*** = **10.6; *P *= 0.002;** $\eta_{G}^{2}$ = **0.16**
		Male	**31.7 (27.6, 35.7)**	45.8 (39.6, 52.0)	**69.4 (58.3, 80.5)**		
	0.10 Hz	Female	**39.0 (28.7, 49.2)**	57.3 (48.1, 66.6)	**84.6 (60.6, 109)**	***F*** = **29.1; *P <* 0.001;** $\eta_{G}^{2}$ = **0.51**	**F** = **7.8; *P* = 0.007;** $\eta_{G}^{2}$ = **0.12**
		Male	**31.4 (26.5, 36.3)**	44.8 (39.6, 49.9)	**67.7 (57.5, 77.8)**		
Mean MCAv ((cm/s)^2^/Hz)	0.05 Hz	Female	**62.2 (49.6, 74.9)**	85.3 (71.1, 99.6)	**117 (88.5, 146)**	***F*** = **28.5; *P <* 0.001;** $\eta_{G}^{2}$ = **0.50**	**F** = **8.8; *P *= 0.004;** $\eta_{G}^{2}$ = **0.14**
		Male	**49.9 (44.7, 55.2)**	68.0 (59.2, 76.8)	**97.3 (83.7, 111)**		
	0.10 Hz	Female	**62.1 (49.6, 74.6)**	84.3 (72.0, 96.6)	**118 (87.7, 148)**	***F*** = **27.3; *P <* 0.001;** $\eta_{G}^{2}$ = **0.49**	***F*** = **7.8; *P *= 0.007;** $\eta_{G}^{2}$ = **0.12**
		Male	**52.5 (45.8, 59.2)**	67.6 (59.8, 75.5)	**97.0 (84.4, 110)**		
Systolic MCAv ((cm/s)^2^/Hz)	0.05 Hz	Female	**97.6 (82.1, 113)**	130 (112, 148)	**168 (135, 201)**	***F*** = **27.9; *P <* 0.001;** $\eta_{G}^{2}$ = **0.50**	***F*** = **4.2; *P *= 0.044;** $\eta_{G}^{2}$ = **0.07**
		Male	**89.3 (78.0, 101)**	114 (98.1, 130)	**149 (130, 167)**		
	0.10 Hz	Female	**104 (88.0, 120)**	133 (117, 149)	**169 (134, 204)**	**F** = **23.7; *P <* 0.001;** $\eta_{G}^{2}$ = **0.46**	**F** = **4.1; *P *= 0.047;** $\eta_{G}^{2}$ = **0.07**
		Male	**95.2 (81.0, 109)**	115 (99.4, 130)	**152 (133, 170)**		
Diastolic PCAv ((cm/s)^2^/Hz)	0.05 Hz	Female	**22.3 (14.7, 29.9)**	34.3 (24.7, 44.0)	**47.3 (31.4, 63.2)**	***F*** = **18.9; *P <* 0.001;** $\eta_{G}^{2}$ = **0.40**	***F*** = **10.5; *P *= 0.002;** $\eta_{G}^{2}$ = **0.16**
		Male	**12.9 (7.50, 18.4)**	22.2 (15.7, 28.7)	**37.0 (31.1, 42.8)**		
	0.10 Hz	Female	**16.5 (8.17, 24.9)**	29.9 (20.1, 39.6)	**45.9 (29.4, 62.5)**	***F*** = **18.6; *P <* 0.001;** $\eta_{G}^{2}$ = **0.40**	***F*** = **6.5; *P *= 0.014;** $\eta_{G}^{2}$ = **0.10**
		Male	**10.6 (6.34, 14.9)**	21.3 (15.0, 27.6)	**33.6 (24.4, 42.8)**		
Mean PCAv ((cm/s)^2^/Hz)	0.05 Hz	Female	**35.5 (26.8, 44.2)**	49.4 (36.5, 62.3)	**64.5 (44.9, 84.0)**	***F*** = **16.4; *P <* 0.001;** $\eta_{G}^{2}$ = **0.37**	***F*** = **7.9; *P *= 0.007;** $\eta_{G}^{2}$ = **0.12**
		Male	**25.9 (20.1, 31.8)**	36.0 (28.6, 43.4)	**53.4 (45.4, 61.3)**		
	0.10 Hz	Female	**34.7 (26.3, 43.0)**	47.9 (35.9, 59.9)	**65.5 (45.0, 86.0)**	***F*** = **15.6; *P <* 0.001;** $\eta_{G}^{2}$ = **0.36**	***F*** = **7.2; *P *= 0.010;** $\eta_{G}^{2}$ = **0.11**
		Male	**26.1 (20.5, 31.8)**	37.1 (30.3, 43.8)	**51.6 (41.6, 61.5)**		
Systolic PCAv ((cm/s)^2^/Hz)	0.05 Hz	Female	**55.2 (44.3, 66.2)**	73.6 (57.0, 90.2)	**93.3 (72.0, 115)**	***F*** = **16.6; *P <* 0.001;** $\eta_{G}^{2}$ = **0.37**	***F*** = **4.4; *P *= 0.040;** $\eta_{G}^{2}$ = **0.07**
		Male	**48.2 (39.0, 57.5)**	60.6 (49.6, 71.6)	**81.5 (68.6, 94.4)**		
	0.10 Hz	Female	**58.2 (48.0, 68.4)**	74.7 (59.0, 90.4)	**96.8 (73.4, 120)**	***F*** = **15.6; *P <* 0.001;** $\eta_{G}^{2}$ = **0.36**	***F*** = **4.8; *P *= 0.032;** $\eta_{G}^{2}$ = **0.08**
		Male	**50.3 (41.4, 59.2)**	62.6 (51.9, 73.4)	**82.5 (68.7, 96.4)**		
Diastolic BP (mmHg)	0.05 Hz	Female	61.4 (53.6, 69.2)	67.2 (59.3, 75.1)	**72.1 (62.3, 82.0)**	*F *= 1.3; *P *= 0.268; $\eta_{G}^{2}$ = 0.05	*F *= 0.7; *P *= 0.411; $\eta_{G}^{2}$ = 0.01
		Male	61.9 (47.8, 76.1)	62.0 (49.8, 74.3)	**66.9 (53.8, 80.1)**		
	0.10 Hz	Female	62.7 (54.7, 70.8)	67.2 (58.9, 75.6)	**66.6 (56.5, 76.8)**	*F *= 0.1; *P *= 0.876; $\eta_{G}^{2}$ = 0.00	*F *= 0.3; *P *= 0.558; $\eta_{G}^{2}$ = 0.01
		Male	63.2 (50.6, 75.9)	63.2 (51.6, 74.7)	**63.4 (49.7, 77.0)**		
MAP (mmHg)	0.05 Hz	Female	81.0 (73.2, 88.9)	88.1 (79.1, 97.0)	**96.0 (85.3, 107)**	*F *= 2.6; *P *= 0.079; $\eta_{G}^{2}$ = 0.09	*F *= 0.2; *P *= 0.638; $\eta_{G}^{2}$ = 0.00
		Male	82.4 (66.8, 97.9)	84.3 (69.9, 98.7)	**92.1 (76.8, 107)**		
	0.10 Hz	Female	83.2 (74.6, 91.8)	88.9 (79.2, 98.6)	**90.4 (81.0, 99.7)**	*F *= 0.4; *P *= 0.649; $\eta_{G}^{2}$ = 0.01	*F *= 0.1; *P *= 0.825; $\eta_{G}^{2}$ = 0.00
		Male	85.5 (72.1, 98.9)	86.1 (72.6, 99.6)	**88.0 (72.1, 104)**		
Systolic BP (mmHg)	0.05 Hz	Female	**126 (111, 141)**	139 (122, 157)	**158 (140, 176)**	***F*** = **6.2; *P *= 0.004;** $\eta_{G}^{2}$ = **0.18**	*F *= 1.6; *P *= 0.210; $\eta_{G}^{2}$ = 0.03
		Male	**137 (116, 159)**	147 (126, 168)	**165 (141, 190)**		
	0.10 Hz	Female	131 (117, 145)	143 (124, 162)	150 (135, 164)	***F*** = **1.7; *P *= 0.200;** $\eta_{G}^{2}$ = **0.06**	*F *= 2.3; *P *= 0.136; $\eta_{G}^{2}$ = 0.04
		Male	146 (129, 164)	150 (130, 170)	158 (131, 185)		
Heart rate (beats/min)	0.05 Hz	Female	93.8 (83.9, 104)	95.3 (86.1, 105)	102 (91.5, 113)	*F *= 1.1; *P *= 0.326; $\eta_{G}^{2}$ = 0.04	***F*** = **6.4; *P *= 0.015;** $\eta_{G}^{2}$ = **0.10**
		Male	89.7 (80.2, 99.1)	86.8 (80.5, 93.1)	90.6 (83.2, 98.1)		
	0.10 Hz	Female	84.9 (76.4, 93.4)	91.4 (81.0, 102)	93.9 (85.2, 103)	*F *= 2.3; *P *= 0.113; $\eta_{G}^{2}$ = 0.07	*F *= 3.1; *p *= 0.083; $\eta_{G}^{2}$ = 0.05
		Male	82.2 (75.6, 88.8)	85.1 (79.4, 90.7)	87.9 (80.6, 95.2)		

*Note*: Data were analysed using analysis of covariance with Dunnett's *post hoc* comparisons comparing hypocapnia and hypercapnia to eucapnia. Hypocapnia and Hypercapnia bold results display a metric that differed from eucapnia, while the latter two columns display group and sex omnibus differences. Data are displayed as means (95% confidence interval). MCAv, middle cerebral artery velocity; PCAv, posterior cerebral artery velocity; $P_{\mathrm{ETC}O_{2}}$, end‐tidal carbon dioxide partial pressure.

**TABLE 2:** Linear mixed‐effects model using participants as random effect outputs for all transfer function analysis variables in 20 participants (10 females and 10 males) including stage (eucapnia as reference) and sex (female and reference).

Vessel	Frequency	Hypocapnia	Hypercapnia	Sex
PSD				
Diastolic BP (mmHg^2^/Hz)	0.05 Hz	374 (5009, 10647); *P *= 0.786; LR *P* = 0.119	−2007 (3435, 5336); *P* = 0.150; LR *P* = 0.119	2591 (−4682, 668); *P* = 0.155; LR *P* = 0.119
	0.10 Hz	2829 (3320, 13237); *P* = 0.292; LR *P* = 0.312	−24 (6649, 10331); *P* = 0.993; LR *P* = 0.312	4024 (−5202, 5155); *P* = 0.188; LR *P* = 0.312
Diastolic MCA ((cm/s)^2^/Hz)	0.05 Hz	−2354 (5071, 11504); *P* = 0.065; LR *P* = 0.034	1327 (3115, 4839); *P* = 0.291; LR *P* = 0.034	707 (−1099, 3753); *P* = 0.743; LR *P* = 0.034
	0.10 Hz	8928 (4048, 26831); *P* = 0.175; LR *P* = 0.546	3213 (16232, 25143); *P* = 0.622; LR *P* = 0.546	2139 (−9429, 15856); *P* = 0.747; LR *P* = 0.546
Diastolic PCA ((cm/s)^2^/Hz)	0.05 Hz	−789 (2157, 5945); *P* = 0.507; LR *P* = 0.416	−1045 (2961, 4425); *P* = 0.381; LR *P* = 0.416	1374 (−3346, 1256); *P* = 0.191; LR *P* = 0.416
	0.10 Hz	5369 (805, 16347); *P* = 0.247; LR *P* = 0.455	−1314 (11477, 17613); *P* = 0.775; LR *P* = 0.455	1660 (−10253, 7625); *P* = 0.707; LR *P* = 0.455
Mean BP (mmHg^2^/Hz)	0.05 Hz	−2400 (9885, 19735); *P* = 0.302; LR *P* = 0.040	−**5237 (5765, 8956); *P* = 0.028; LR *P* = 0.040**	5441 (−9727, −748); *P* = 0.096; LR *P* = 0.040
	0.10 Hz	2092 (7236, 18767); *P* = 0.376; LR *P* = 0.705	1518 (5866, 9113); *P* = 0.519; LR *P* = 0.705	2566 (−3050, 6087); *P* = 0.505; LR *P* = 0.705
Mean MCA ((cm/s)^2^/Hz)	0.05 Hz	−**6763 (7816, 15121); *P* < 0.001; LR *P* < 0.001**	1197 (3946, 6130); *P* = 0.451; LR *P <* 0.001	−474 (−1877, 4270); *P* = 0.843; LR *P <* 0.001
	0.10 Hz	−85 (10767, 24490); *P* = 0.966; LR *P* = 0.392	2884 (5029, 7813); *P* = 0.158; LR *P* = 0.392	−1437 (−1033, 6801); *P* = 0.765; LR *P* = 0.392
Mean PCA ((cm/s)^2^/Hz)	0.05 Hz	−**1844 (2113, 5219); *P* = 0.011; LR *P* = 0.012**	189 (1737, 2699); *P* = 0.786; LR *P* = 0.012	1046 (−1164, 1542); *P* = 0.304; LR *P* = 0.012
	0.10 Hz	767 (3894, 10142); *P* = 0.536; LR *P* = 0.851	977 (3090, 4801); *P* = 0.432; LR *P* = 0.851	−490 (−1430, 3384); *P* = 0.814; LR *P* = 0.851
Systolic BP (mmHg^2^/Hz)	0.05 Hz	−2384 (10300, 25148); *P* = 0.481; LR *P* = 0.068	−5411 (8417, 13076); *P* = 0.114; LR *P* = 0.068	**9996 (**−**11966, 1144); *P* = 0.048; LR *P* = 0.068**
	0.10 Hz	−3013 (5706, 24550); *P* = 0.268; LR *P* = 0.297	−1340 (6740, 10471); *P* = 0.620; LR *P* = 0.297	9809 (−6589, 3909); *P* = 0.149; LR *P* = 0.297
Systolic MCA ((cm/s)^2^/Hz)	0.05 Hz	−1831 (2177, 5555); *P* = 0.060; LR *P* = 0.009	1364 (2372, 3682); *P* = 0.157; LR *P* = 0.009	862 (−483, 3211); *P* = 0.389; LR *P* = 0.009
	0.10 Hz	331 (2024, 9882); *P* = 0.822; LR *P* = 0.422	2239 (3668, 5699); *P* = 0.133; LR *P* = 0.422	−394 (−618, 5096); *P* = 0.882; LR *P* = 0.422
Systolic PCA ((cm/s)^2^/Hz)	0.05 Hz	−956 (818, 2373); *P* = 0.059; LR *P* = 0.034	471 (1285, 1829); *P* = 0.343; LR *P* = 0.034	122 (−481, 1424); *P* = 0.770; LR *P* = 0.034
	0.10 Hz	−894 (1473, 4238); *P* = 0.282; LR *P* = 0.059	1093 (2056, 3147); *P* = 0.190; LR *P* = 0.059	−961 (−509, 2695); *P* = 0.227; LR *P* = 0.059
Coherence				
Diastolic MCA ((cm/s)^2^/Hz)	0.05 Hz	−0.01 (0.90, 0.99); *P* = 0.815; LR *P* = 0.152	−0.05 (0.07, 0.11); *P* = 0.055; LR *P* = 0.152	0.02 (−0.11, −0.00); *P* = 0.461; LR *P* = 0.152
	0.10 Hz	−0.01 (0.94, 1.02); *P* = 0.530; LR *P* = 0.741	−0.01 (0.06, 0.09); *P* = 0.544; LR *P* = 0.741	−0.02 (−0.06, 0.03); *P* = 0.431; LR *P* = 0.741
Diastolic PCA ((cm/s)^2^/Hz)	0.05 Hz	0.03 (0.88, 0.95); *P* = 0.176; LR *P* = 0.173	−0.01 (0.05, 0.07); *P* = 0.586; LR *P* = 0.173	0.02 (−0.05, 0.03); *P* = 0.343; LR *P* = 0.173
	0.10 Hz	0.01 (0.93, 1.00); *P* = 0.586; LR *P* = 0.437	−0.02 (0.05, 0.08); *P* = 0.324; LR *P* = 0.437	−0.01 (−0.06, 0.02); *P* = 0.629; LR *P* = 0.437
Mean MCA ((cm/s)^2^/Hz)	0.05 Hz	−0.02 (0.91, 0.98); *P* = 0.295; LR *P* = 0.192	−0.03 (0.05, 0.08); *P* = 0.180; LR *P* = 0.192	0.03 (−0.07, 0.01); *P* = 0.129; LR *P* = 0.192
	0.10 Hz	−0.01 (0.97, 1.00); *P* = 0.146; LR *P* = 0.376	−0.00 (0.02, 0.03); *P* = 0.932; LR *P* = 0.376	−0.00 (−0.02, 0.02); *P* = 0.617; LR *P* = 0.376
Mean PCA ((cm/s)^2^/Hz)	0.05 Hz	−0.01 (0.92, 0.98); *P* = 0.552; LR *P* = 0.264	−0.02 (0.04, 0.06); *P* = 0.139; LR *P* = 0.264	0.02 (−0.06, 0.01); *P* = 0.234; LR *P* = 0.264
	0.10 Hz	−0.01 (0.95, 1.03); *P* = 0.470; LR *P* = 0.321	−0.03 (0.05, 0.08); *P* = 0.118; LR *P* = 0.321	−0.02 (−0.07, 0.01); *P* = 0.379; LR *P* = 0.321
Systolic MCA ((cm/s)^2^/Hz)	0.05 Hz	0.02 (0.76, 0.91); *P* = 0.736; LR *P* = 0.636	−0.03 (0.12, 0.17); *P* = 0.479; LR *P* = 0.636	0.03 (−0.13, 0.06); *P* = 0.497; LR *P* = 0.636
	0.10 Hz	−0.02 (0.92, 0.99); *P* = 0.219; LR *P* = 0.166	−0.02 (0.04, 0.06); *P* = 0.160; LR *P* = 0.166	−0.04 (−0.06, 0.01); *P* = 0.134; LR *P* = 0.166
Systolic PCA ((cm/s)^2^/Hz)	0.05 Hz	0.00 (0.76, 0.89); *P* = 0.931; LR *P* = 0.698	−0.02 (0.08, 0.13); *P* = 0.574; LR *P* = 0.698	0.04 (−0.09, 0.05); *P* = 0.353; LR *P* = 0.698
	0.10 Hz	−**0.06 (0.92, 1.01); *P* = 0.018; LR *P* = 0.028**	−0.05 (0.06, 0.09); *P* = 0.067; LR *P* = 0.028	−0.04 (−0.10, 0.00); *P* = 0.143; LR *P* = 0.028
Phase (radians)				
Diastolic MCA ((cm/s)^2^/Hz)	0.05 Hz	**0.16 (0.40, 0.65); *P* = 0.037; LR *P* < 0.001**	−**0.30 (0.19, 0.29); *P* < 0.001; LR *P* < 0.001**	−0.00 (−0.44, −0.15); *P* = 0.981; LR *P <* 0.001
	0.10 Hz	**0.14 (0.29, 0.46); *P* = 0.006; LR *P* < 0.001**	−**0.14 (0.12, 0.19); *P* = 0.006; LR *P* < 0.001**	0.00 (−0.24, −0.05); *P* = 0.931; LR *P <* 0.001
Diastolic PCA ((cm/s)^2^/Hz)	0.05 Hz	**0.18 (0.29, 0.60); *P* = 0.027; LR *P* < 0.001**	−**0.22 (0.16, 0.26); *P* = 0.006; LR *P* < 0.001**	0.01 (−0.37, −0.07); *P* = 0.935; LR *P <* 0.001
	0.10 Hz	0.07 (0.30, 0.53); *P* = 0.337; LR *P* = 0.079	−0.10 (0.16, 0.25); *P* = 0.132; LR *P* = 0.079	−0.00 (−0.23, 0.03); *P* = 0.991; LR *P* = 0.079
Mean MCA ((cm/s)^2^/Hz)	0.05 Hz	**0.38 (0.33, 0.55); *P* < 0.001; LR *P* < 0.001**	−**0.28 (0.16, 0.24); *P* < 0.001; LR *P* < 0.001**	0.05 (−0.40, −0.16); *P* = 0.478; LR *P <* 0.001
	0.10 Hz	**0.20 (0.30, 0.45); *P* < 0.001; LR *P* < 0.001**	−**0.20 (0.11, 0.16); *P* < 0.001; LR *P* < 0.001**	0.05 (−0.28, −0.12); *P* = 0.283; LR *P <* 0.001
Mean PCA ((cm/s)^2^/Hz)	0.05 Hz	**0.30 (0.31, 0.56); *P* < 0.001; LR *P* < 0.001**	−**0.24 (0.17, 0.26); *P* =** **0.001; LR *P* < 0.001**	−0.01 (−0.37, −0.11); *P* = 0.889; LR *P <* 0.001
	0.10 Hz	**0.18 (0.28, 0.45); *P* < 0.001; LR *P* < 0.001**	−**0.17 (0.09, 0.14); *P* < 0.001; LR *P* < 0.001**	0.07 (−0.25, −0.10); *P* = 0.234; LR *P <* 0.001
Systolic MCA ((cm/s)^2^/Hz)	0.05 Hz	**1.06 (0.52, 1.07); *P* < 0.001; LR *P* < 0.001**	−**0.48 (0.36, 0.57); *P* = 0.002; LR *P* < 0.001**	0.22 (−0.76, −0.20); *P* = 0.190; LR *P <* 0.001
	0.10 Hz	**0.42 (0.55, 1.11); *P* = 0.022; LR *P *= 0.001**	−0.35 (0.46, 0.65); P = 0.055; LR P = 0.001	0.01 (−0.68, −0.01); *P* = 0.924; LR *P* = 0.001
Systolic PCA ((cm/s)^2^/Hz)	0.05 Hz	**0.90 (0.56, 1.41); *P* < 0.001; LR *P* < 0.001**	−**0.58 (0.53, 0.83); *P* = 0.009; LR *P* < 0.001**	−0.11 (−1.00, −0.17); *P* = 0.682; LR *P <* 0.001
	0.10 Hz	**0.43 (0.48, 1.08); P = 0.026; LR *P* < 0.001**	−**0.58 (0.49, 0.70); *P* = 0.003; LR *P* < 0.001**	0.22 (−0.95, −0.22); *P* = 0.160; LR *P <* 0.001
Gain (cm/s/mmHg)				
Diastolic MCA ((cm/s)^2^/Hz)	0.05 Hz	−**0.22 (0.84, 1.14); *P* = 0.001; LR *P *< 0.001**	**0.13 (0.16, 0.24); *P* = 0.039; LR *P* < 0.001**	−0.00 (0.01, 0.25); *P* = 0.964; LR *P <* 0.001
	0.10 Hz	−0.00 (1.17, 1.53); *P* = 0.994; LR *P* = 0.171	0.10 (0.18, 0.28); *P* = 0.187; LR *P* = 0.171	−0.18 (−0.04, 0.24); *P* = 0.136; LR *P* = 0.171
Diastolic PCA ((cm/s)^2^/Hz)	0.05 Hz	−0.07 (0.56, 0.80); *P* = 0.274; LR *P* = 0.676	−0.01 (0.15, 0.24); *P* = 0.900; LR *P* = 0.676	0.00 (−0.13, 0.11); *P* = 0.947; LR *P* = 0.676
	0.10 Hz	0.04 (0.88, 1.17); *P* = 0.643; LR *P* = 0.043	−0.13 (0.19, 0.30); *P* = 0.112; LR *P* = 0.043	−0.16 (−0.28, 0.03); *P* = 0.087; LR *P* = 0.043
Mean MCA ((cm/s)^2^/Hz)	0.05 Hz	−**0.25 (0.72, 0.95); *P* < 0.001; LR *P* < 0.001**	**0.20 (0.12, 0.19); *P* < 0.001; LR *P* < 0.001**	−0.10 (0.10, 0.30); *P* = 0.205; LR *P <* 0.001
	0.10 Hz	−**0.12 (1.02, 1.27); *P* = 0.041; LR *P* = 0.034**	0.01 (0.14, 0.22); *P* = 0.838; LR *P* = 0.034	−0.12 (−0.10, 0.12); *P* = 0.160; LR *P* = 0.034
Mean PCA ((cm/s)^2^/Hz)	0.05 Hz	−**0.11 (0.41, 0.58); *P* = 0.003; LR *P* < 0.001**	0.06 (0.09, 0.13); *P* = 0.065; LR *P <* 0.001	−0.01 (−0.00, 0.13); *P* = 0.855; LR *P <* 0.001
	0.10 Hz	−0.03 (0.62, 0.84); *P* = 0.599; LR *P* = 0.516	−0.05 (0.12, 0.19); *P* = 0.319; LR *P* = 0.516	−0.08 (−0.14, 0.05); *P* = 0.301; LR *P* = 0.516
Systolic MCA ((cm/s)^2^/Hz)	0.05 Hz	−0.04 (0.34, 0.58); *P* = 0.507; LR *P* = 0.224	0.08 (0.15, 0.24); *P* = 0.227; LR *P* = 0.224	−0.06 (−0.04, 0.20); *P* = 0.451; LR *P* = 0.224
	0.10 Hz	0.05 (0.54, 0.79); *P* = 0.494; LR *P* = 0.032	0.04 (0.17, 0.27); *P* = 0.559; LR *P* = 0.032	−**0.23 (**−**0.10, 0.18); P = 0.007; LR P = 0.032**
Systolic PCA ((cm/s)^2^/Hz)	0.05 Hz	−0.08 (0.22, 0.41); *P* = 0.089; LR *P* = 0.082	0.02 (0.11, 0.17); *P* = 0.665; LR *P* = 0.082	−0.07 (−0.07, 0.11); *P* = 0.258; LR *P* = 0.082
	0.10 Hz	−0.08 (0.36, 0.56); *P* = 0.111; LR *P* = 0.015	0.01 (0.12, 0.19); *P* = 0.910; LR *P* = 0.015	−**0.17 (**−**0.09, 0.10); P = 0.016; LR P = 0.015**
nGain				
Diastolic MCA ((cm/s)^2^/Hz)	0.05 Hz	0.24 (1.32, 1.98); *P* = 0.146; LR *P* = 0.001	−**0.35 (0.41, 0.64); *P* = 0.039; LR *P* = 0.001**	**0.50 (**−**0.67,** −**0.03); *P* = 0.025; LR *P* = 0.001**
	0.10 Hz	**1.13 (1.82, 2.81); *P* < 0.001; LR *P* < 0.001**	−**0.56 (0.54, 0.85); *P* = 0.013; LR *P* < 0.001**	0.27 (−0.99, −0.14); *P* = 0.412; LR *P <* 0.001
Diastolic PCA ((cm/s)^2^/Hz)	0.05 Hz	**1.76 (1.09, 3.11); *P* = 0.001; LR *P* < 0.001**	−**1.14 (1.28, 1.98); *P* = 0.031; LR *P* < 0.001**	**1.53 (**−**2.13,** −**0.15); *P* = 0.023; LR *P* < 0.001**
	0.10 Hz	**2.49 (2.47, 4.84); P < 0.001; LR *P* < 0.001**	−**1.93 (1.22, 1.90); *P* < 0.001; LR *P* < 0.001**	0.89 (−2.89, −0.98); *P* = 0.264; LR *P <* 0.001
Mean MCA ((cm/s)^2^/Hz)	0.05 Hz	−0.11 (0.92, 1.14); *P* = 0.098; LR *P* = 0.071	−0.12 (0.16, 0.25); *P* = 0.064; LR *P* = 0.071	0.09 (−0.25, 0.00); *P* = 0.127; LR *P* = 0.071
	0.10 Hz	**0.29 (1.19, 1.59); *P* = 0.001; LR *P* < 0.001**	−**0.35 (0.21, 0.33); *P* < 0.001; LR *P* < 0.001**	0.08 (−0.52, −0.19); *P* = 0.527; LR *P <* 0.001
Mean PCA ((cm/s)^2^/Hz)	0.05 Hz	0.12 (0.81, 1.35); *P* = 0.305; LR *P* = 0.002	−**0.27 (0.28, 0.44); *P* = 0.023; LR *P* = 0.002**	0.32 (−0.48, −0.05); *P* = 0.078; LR *P* = 0.002
	0.10 Hz	**0.64 (1.15, 1.97); *P* < 0.001; LR *P* < 0.001**	−**0.55 (0.41, 0.64); *P* = 0.002; LR *P* < 0.001**	0.31 (−0.87, −0.23); *P* = 0.268; LR *P <* 0.001
Systolic MCA ((cm/s)^2^/Hz)	0.05 Hz	0.06 (0.27, 0.46); *P* = 0.222; LR *P* = 0.395	−0.02 (0.12, 0.19); *P* = 0.697; LR *P* = 0.395	−0.01 (−0.11, 0.08); *P* = 0.876; LR *P* = 0.395
	0.10 Hz	**0.17 (0.42, 0.65); *P* = 0.007; LR *P* = 0.001**	−0.04 (0.15, 0.24); *P* = 0.512; LR *P* = 0.001	−**0.15 (**−**0.16, 0.08); *P* = 0.041; LR *P* = 0.001**
Systolic PCA ((cm/s)^2^/Hz)	0.05 Hz	−0.02 (0.32, 0.51); *P* = 0.757; LR *P* = 0.850	−0.02 (0.13, 0.20); *P* = 0.625; LR *P* = 0.850	−0.04 (−0.12, 0.07); *P* = 0.496; LR *P* = 0.850
	0.10 Hz	−0.00 (0.51, 0.79); *P* = 0.997; LR *P* = 0.136	−0.10 (0.18, 0.29); *P* = 0.175; LR *P* = 0.136	−0.15 (−0.24, 0.04); *P* = 0.107; LR *P* = 0.136

*Note*: Significant differences are denoted in bold font. Data are displayed as means (95% confidence interval). *P‐*value, likelihood ratio test *P*‐value. Abbreviations: BP, blood pressure; MCA, middle cerebral artery; nGain, normalized gain; PCA, posterior cerebral artery; PSD, power spectral density.

**TABLE 3:** Absolute transfer functional analysis estimates observed during 0.05 and 0.10 Hz repeated squat–stand manoeuvres across three separate stages, hypocapnia, eucapnia (reference) and hypercapnia, in 20 participants (10 females and 10 males).

Component	Frequency	Hypocapnia	Eucapnia	Hypercapnia
PSD				
Diastolic				
BP (mmHg^2^/Hz)	0.05 Hz	9498 (6553, 12442)	9123 (6749, 11497)	7116 (4991, 9241)
	0.10 Hz	13119 (6417, 19821)	10290 (7538, 13043)	10267 (7459, 13075)
MCAv ((cm/s)^2^/Hz)	0.05 Hz	6287 (4276, 8297)	8641 (6185, 11096)	9968 (6681, 13255)
	0.10 Hz	25438 (9246, 41630)	16509 (11795, 21224)	19722 (14100, 25345)
PCAv ((cm/s)^2^/Hz)	0.05 Hz	3949 (2149, 5749)	4738 (2587, 6890)	3531 (2190, 4873)
	0.10 Hz	14775 (3073, 26477)	9406 (6808, 12004)	8502 (5879, 11125)
Mean				
BP (mmHg^2^/Hz)	0.05 Hz	15131 (10266, 19995)	17531 (12644, 22418)	12293 (9068, 15519)
	0.10 Hz	16377 (10992, 21762)	14284 (10120, 18449)	15803 (11022, 20583)
MCAv ((cm/s)^2^/Hz)	0.05 Hz	4469 (3115, 5822)	11232 (7202, 15262)	12428 (9271, 15586)
	0.10 Hz	16825 (11378, 22272)	16910 (11948, 21872)	19794 (14035, 25553)
PCAv ((cm/s)^2^/Hz)	0.05 Hz	2345 (1493, 3197)	4189 (2804, 5574)	4378 (2740, 6017)
	0.10 Hz	7540 (4857, 10223)	6773 (4864, 8682)	7750 (4755, 10744)
Systolic				
BP (mmHg^2^/Hz)	0.05 Hz	20338 (11603, 29073)	22722 (17245, 28199)	17311 (11906, 22716)
	0.10 Hz	17020 (9931, 24108)	20033 (11667, 28399)	18693 (10964, 26422)
MCAv ((cm/s)^2^/Hz)	0.05 Hz	2528 (1833, 3222)	4502 (3028, 5976)	5472 (3151, 7792)
	0.10 Hz	6087 (3157, 9017)	5756 (3533, 7979)	8811 (4356, 13266)
PCAv ((cm/s)^2^/Hz)	0.05 Hz	701 (390, 1012)	1657 (938, 2375)	2428 (1228, 3629)
	0.10 Hz	1481 (945, 2017)	2375 (1560, 3189)	4221 (1841, 6601)
Coherence				
Diastolic				
MCAv ((cm/s)^2^/Hz)	0.05 Hz	0.95 (0.92, 0.98)	0.95 (0.94, 0.97)	0.90 (0.84, 0.96)
	0.10 Hz	0.95 (0.91, 1.00)	0.97 (0.94, 0.99)	0.95 (0.92, 0.99)
PCAv ((cm/s)^2^/Hz)	0.05 Hz	0.95 (0.93, 0.97)	0.92 (0.89, 0.96)	0.91 (0.87, 0.95)
	0.10 Hz	0.97 (0.96, 0.98)	0.96 (0.94, 0.99)	0.94 (0.89, 0.99)
Mean				
MCAv ((cm/s)^2^/Hz)	0.05 Hz	0.94 (0.91, 0.96)	0.96 (0.94, 0.98)	0.93 (0.88, 0.98)
	0.10 Hz	0.97 (0.95, 0.99)	0.98 (0.97, 1.00)	0.98 (0.97, 0.99)
PCAv ((cm/s)^2^/Hz)	0.05 Hz	0.95 (0.93, 0.97)	0.96 (0.94, 0.98)	0.93 (0.90, 0.97)
	0.10 Hz	0.97 (0.95, 0.99)	0.98 (0.97, 0.99)	0.95 (0.90, 1.00)
Systolic				
MCAv ((cm/s)^2^/Hz)	0.05 Hz	0.86 (0.81, 0.92)	0.85 (0.77, 0.92)	0.81 (0.73, 0.89)
	0.10 Hz	0.91 (0.89, 0.94)	0.93 (0.90, 0.96)	0.91 (0.87, 0.95)
PCAv ((cm/s)^2^/Hz)	0.05 Hz	0.85 (0.80, 0.90)	0.85 (0.79, 0.91)	0.83 (0.76, 0.90)
	0.10 Hz	0.89 (0.83, 0.94)	0.95 (0.92, 0.97)	0.90 (0.85, 0.94)
Phase				
Diastolic				
MCAv ((cm/s)^2^/Hz)	0.05 Hz	0.68 (0.53, 0.84)	0.52 (0.43, 0.62)	0.22 (0.13, 0.32)
	0.10 Hz	0.52 (0.44, 0.60)	0.38 (0.30, 0.46)	0.23 (0.16, 0.31)
PCAv ((cm/s)^2^/Hz)	0.05 Hz	0.62 (0.47, 0.78)	0.44 (0.32, 0.56)	0.22 (0.14, 0.31)
	0.10 Hz	0.48 (0.40, 0.56)	0.41 (0.35, 0.48)	0.25 (0.17, 0.34)
Mean				
MCAv ((cm/s)^2^/Hz)	0.05 Hz	0.85 (0.73, 0.96)	0.47 (0.36, 0.57)	0.19 (0.10, 0.27)
	0.10 Hz	0.60 (0.51, 0.69)	0.40 (0.33, 0.46)	0.19 (0.14, 0.25)
PCAv ((cm/s)^2^/Hz)	0.05 Hz	0.73 (0.60, 0.87)	0.43 (0.31, 0.55)	0.19 (0.12, 0.26)
	0.10 Hz	0.58 (0.52, 0.65)	0.40 (0.34, 0.46)	0.23 (0.14, 0.31)
Systolic				
MCAv ((cm/s)^2^/Hz)	0.05 Hz	1.93 (1.62, 2.23)	0.93 (0.68, 1.18)	0.41 (0.19, 0.63)
	0.10 Hz	1.25 (0.98, 1.53)	0.84 (0.69, 0.99)	0.49 (0.39, 0.60)
PCAv ((cm/s)^2^/Hz)	0.05 Hz	1.83 (1.33, 2.34)	0.93 (0.64, 1.23)	0.35 (0.09, 0.61)
	0.10 Hz	1.32 (1.08, 1.56)	0.89 (0.73, 1.05)	0.56 (0.40, 0.73)
Gain				
Diastolic				
MCAv ((cm/s)^2^/Hz)	0.05 Hz	0.77 (0.63, 0.90)	0.98 (0.86, 1.11)	1.12 (1.01, 1.23)
	0.10 Hz	1.26 (1.11, 1.42)	1.26 (1.09, 1.43)	1.36 (1.22, 1.50)
PCAv ((cm/s)^2^/Hz)	0.05 Hz	0.62 (0.53, 0.70)	0.68 (0.57, 0.79)	0.66 (0.55, 0.78)
	0.10 Hz	0.98 (0.84, 1.12)	0.94 (0.81, 1.08)	0.85 (0.74, 0.97)
Mean				
MCAv ((cm/s)^2^/Hz)	0.05 Hz	0.54 (0.46, 0.62)	0.79 (0.69, 0.89)	0.99 (0.88, 1.10)
	0.10 Hz	0.97 (0.86, 1.08)	1.09 (0.97, 1.21)	1.10 (1.01, 1.19)
PCAv ((cm/s)^2^/Hz)	0.05 Hz	0.38 (0.34, 0.43)	0.49 (0.41, 0.57)	0.56 (0.47, 0.64)
	0.10 Hz	0.67 (0.57, 0.76)	0.69 (0.60, 0.79)	0.64 (0.54, 0.74)
Systolic				
MCAv ((cm/s)^2^/Hz)	0.05 Hz	0.40 (0.28, 0.52)	0.46 (0.33, 0.58)	0.53 (0.42, 0.63)
	0.10 Hz	0.60 (0.48, 0.72)	0.55 (0.46, 0.65)	0.65 (0.50, 0.79)
PCAv ((cm/s)^2^/Hz)	0.05 Hz	0.20 (0.13, 0.26)	0.28 (0.17, 0.39)	0.33 (0.26, 0.40)
	0.10 Hz	0.29 (0.23, 0.36)	0.37 (0.29, 0.46)	0.43 (0.28, 0.58)
nGain				
Diastolic				
MCAv ((cm/s)^2^/Hz)	0.05 Hz	2.14 (1.70, 2.58)	1.90 (1.69, 2.10)	1.55 (1.31, 1.79)
	0.10 Hz	3.58 (2.95, 4.20)	2.45 (2.16, 2.74)	1.88 (1.63, 2.13)
PCAv ((cm/s)^2^/Hz)	0.05 Hz	4.62 (3.20, 6.05)	2.87 (2.10, 3.63)	1.63 (1.37, 1.89)
	0.10 Hz	6.59 (5.38, 7.80)	4.10 (2.92, 5.28)	2.22 (1.83, 2.61)
Mean				
MCAv ((cm/s)^2^/Hz)	0.05 Hz	0.97 (0.85, 1.08)	1.08 (0.98, 1.17)	0.95 (0.86, 1.04)
	0.10 Hz	1.73 (1.50, 1.96)	1.43 (1.30, 1.56)	1.08 (0.95, 1.20)
PCAv ((cm/s)^2^/Hz)	0.05 Hz	1.36 (1.10, 1.62)	1.24 (0.96, 1.53)	0.98 (0.85, 1.11)
	0.10 Hz	2.35 (1.90, 2.80)	1.71 (1.38, 2.04)	1.16 (0.94, 1.37)
Systolic				
MCAv ((cm/s)^2^/Hz)	0.05 Hz	0.43 (0.33, 0.53)	0.38 (0.29, 0.46)	0.36 (0.29, 0.43)
	0.10 Hz	0.63 (0.51, 0.75)	0.46 (0.39, 0.52)	0.46 (0.33, 0.59)
PCAv ((cm/s)^2^/Hz)	0.05 Hz	0.38 (0.29, 0.46)	0.39 (0.30, 0.48)	0.41 (0.33, 0.49)
	0.10 Hz	0.58 (0.48, 0.67)	0.58 (0.46, 0.69)	0.51 (0.32, 0.71)

*Note*: Data are displayed as means (95% confidence interval). Sex differences were also compared across all three stages. Abbreviations: BP, blood pressure; MCAv, middle cerebral artery velocity; nGain, normalized gain; PCAv, posterior cerebral artery; PSD, power spectral density.

**FIGURE 3:**
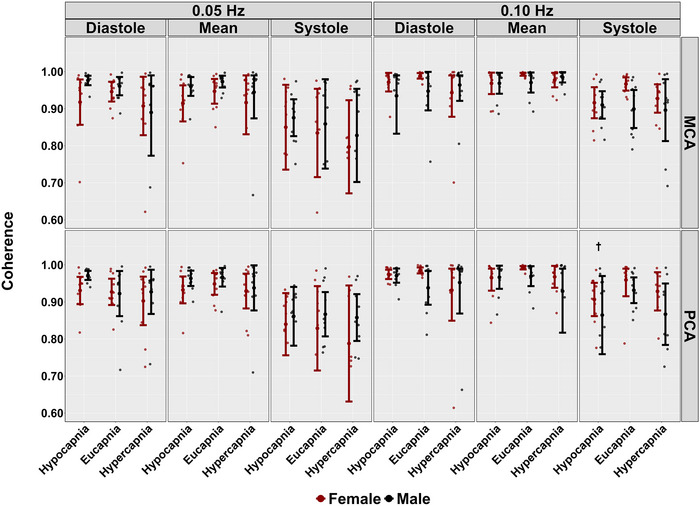
Coherence for middle cerebral artery (MCA) and posterior cerebral artery (PCA) obtained across the cardiac cycle during repeated squat–stand manoeuvres performed at frequencies of 0.05 and 0.10 Hz, across three stages: hypocapnia, eucapnia and hypercapnia. The data are categorized by sex, with females represented in red (*n* = 10) and males in black (*n* = 10). † indicates a stage that was significantly different from the eucapnia stage, as determined by linear regressions with stages (eucapnia as reference) and sex (female as reference) as predictor variables.

**FIGURE 4:**
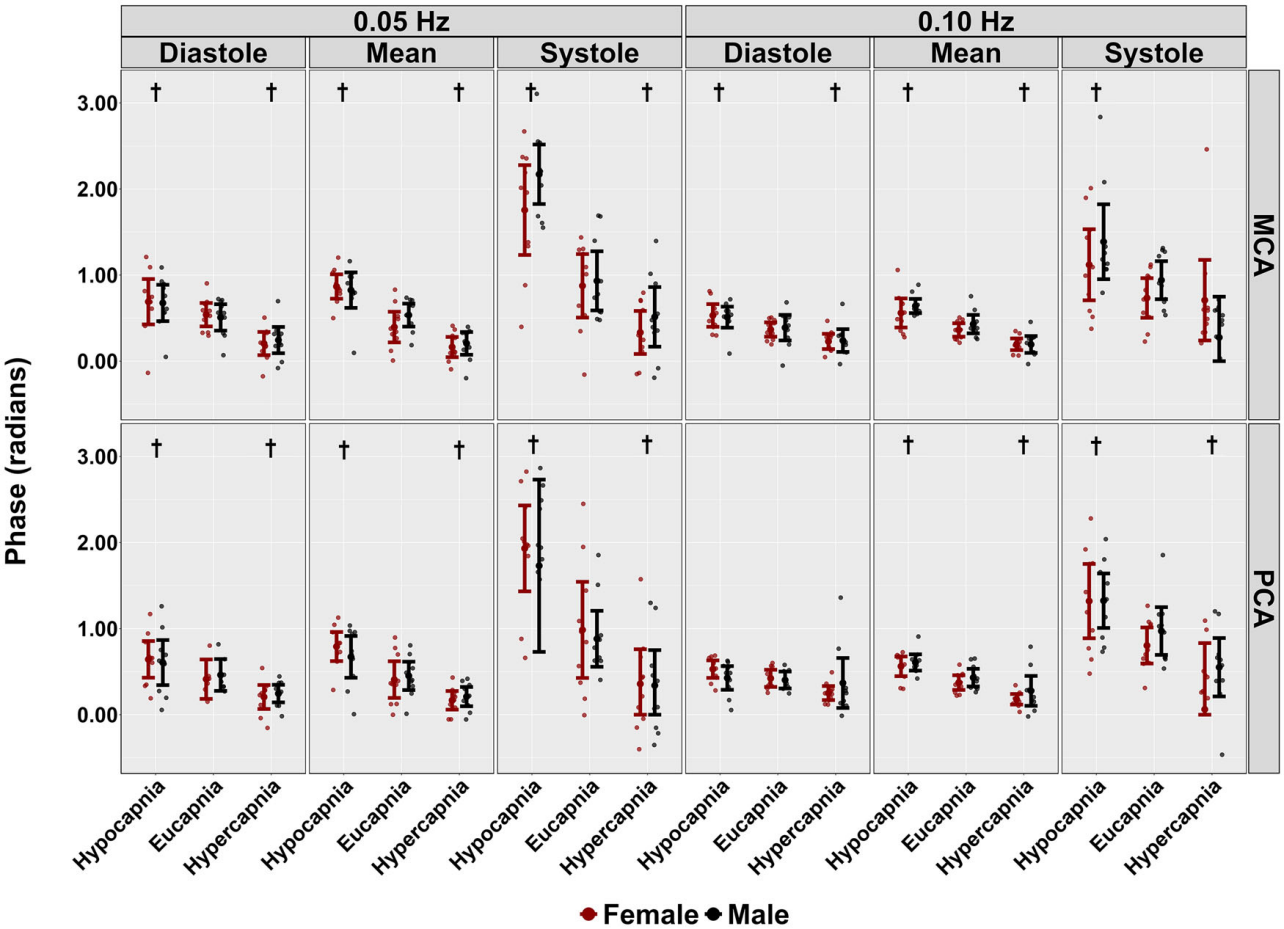
Phase for middle cerebral artery (MCA) and posterior cerebral artery (PCA) obtained across the cardiac cycle during repeated squat–stand manoeuvres performed at frequencies of 0.05 and 0.10 Hz, across three stages: hypocapnia, eucapnia and hypercapnia. The data are categorized by sex, with females represented in red (*n* = 10) and males in black (*n* = 10). † indicates a stage that was significantly different from the eucapnia stage, as determined by linear regressions with stages (eucapnia as reference) and sex (female as reference) as predictor variables.

**FIGURE 5:**
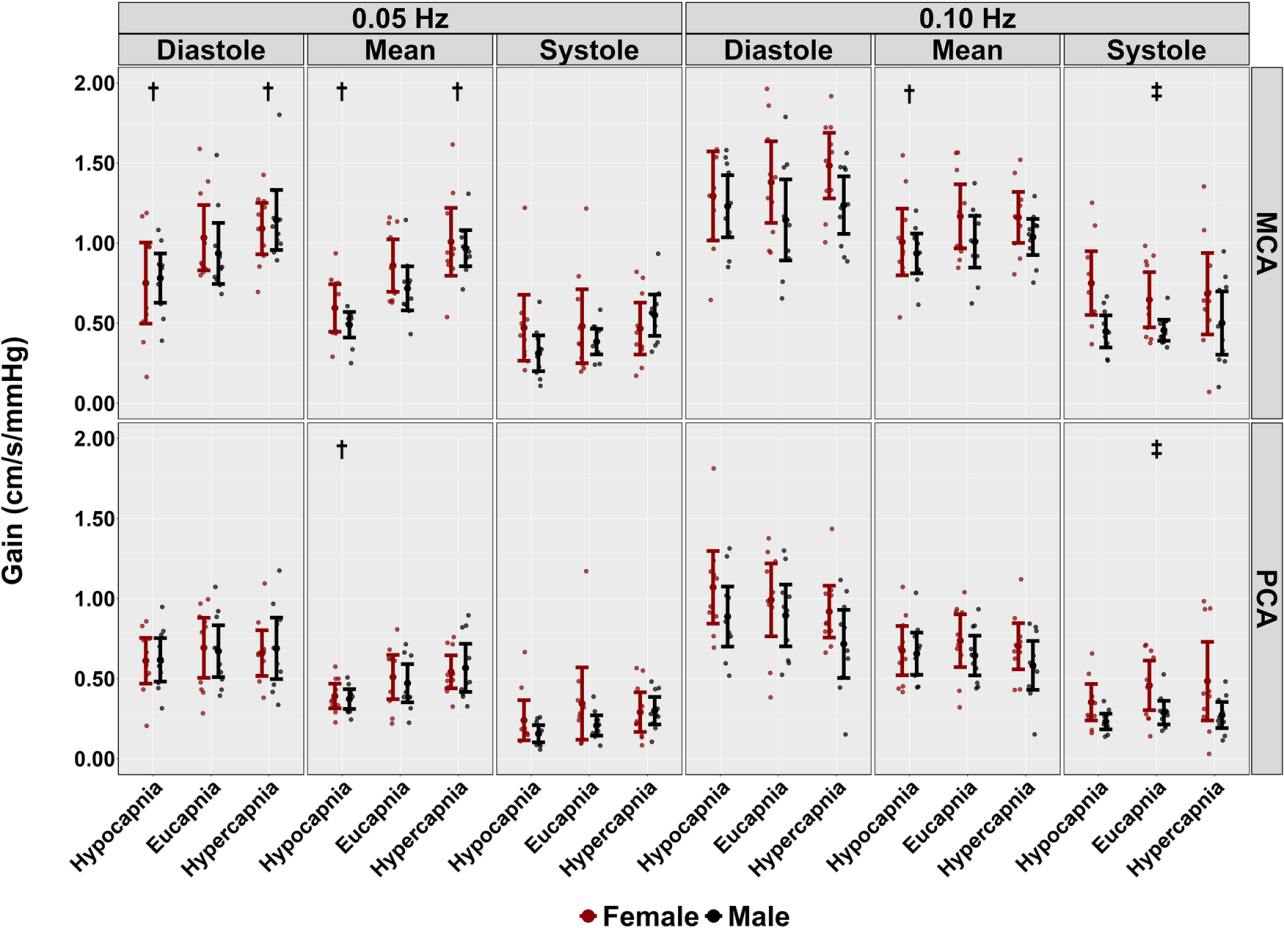
Gain for middle cerebral artery (MCA) and posterior cerebral artery (PCA) obtained across the cardiac cycle during repeated squat–stand manoeuvres performed at frequencies of 0.05 and 0.10 Hz, across three stages: hypocapnia, eucapnia and hypercapnia. The data are categorized by sex, with females represented in red (*n* = 10) and males in black (*n* = 10). † indicates a stage that was significantly different from the eucapnia stage, as determined by linear regressions with stages (eucapnia as reference) and sex (female as reference) as predictor variables. ‡ highlights sex differences observed across the three stages (hypocapnia, eucapnia, hypercapnia).

**FIGURE 6:**
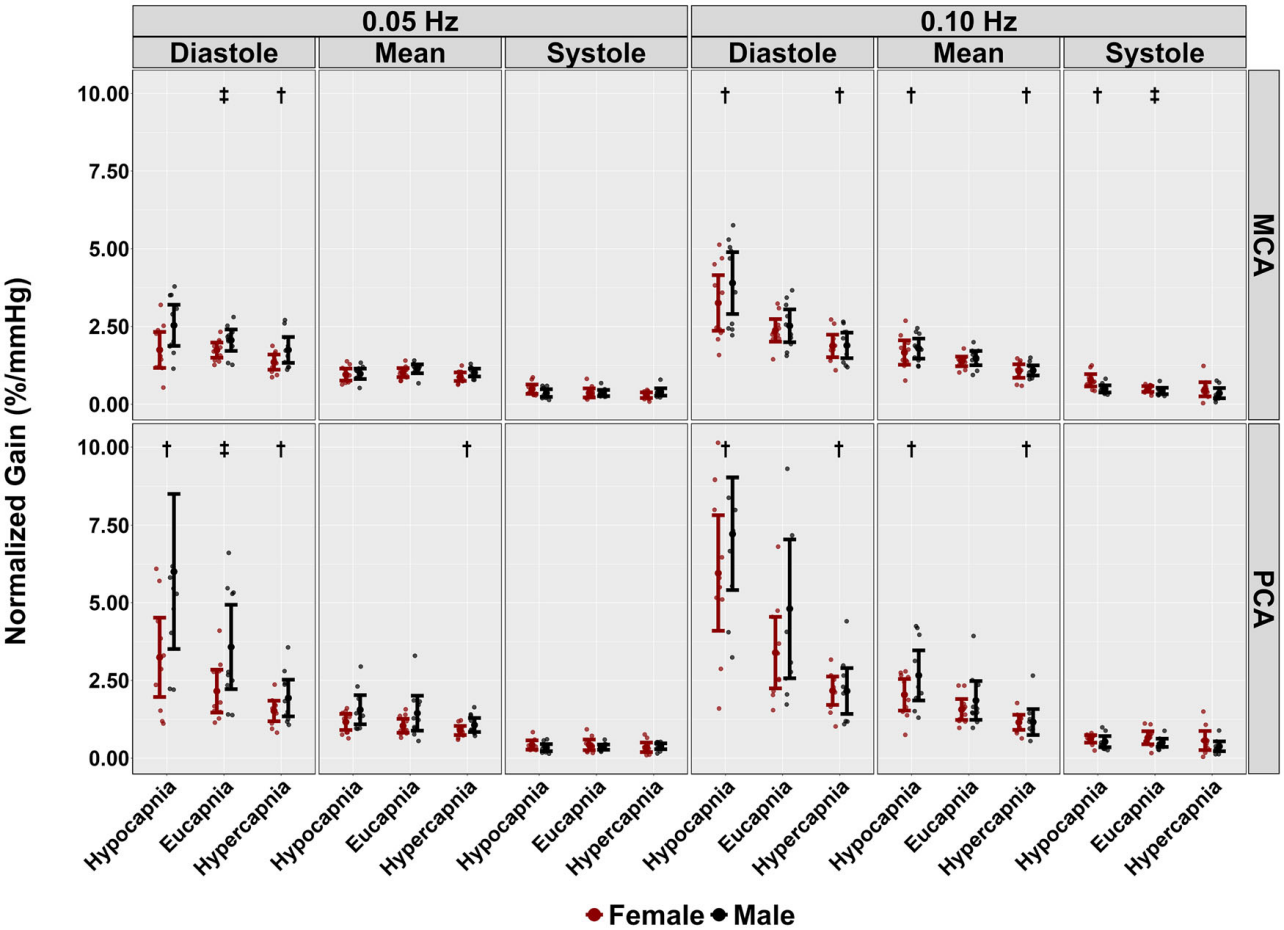
Normalized gain (nGain) for middle cerebral artery (MCA) and posterior cerebral artery (PCA) obtained across the cardiac cycle during repeated squat–stand manoeuvres performed at frequencies of 0.05 and 0.10 Hz, across three stages: hypocapnia, eucapnia and hypercapnia. The data are categorized by sex, with females represented in red (*n* = 10) and males in black (*n* = 10). † indicates a stage that was significantly different from the eucapnia stage, as determined by linear regressions with stages (eucapnia as reference) and sex (female as reference) as predictor variables. ‡ highlights sex differences observed across the three stages (hypocapnia, eucapnia, hypercapnia).

During hypercapnic conditions, no differences were observed across the cardiac cycle in PSD metrics at 0.05 and 0.10 Hz stages (all *P >* 0.114), except for systole mean BP at 0.05 Hz (*P *= 0.028, LR* P *= 0.040) (Table 2). Additionally, across the cardiac cycle, coherence did not differ across both frequencies of SSMs during hypercapnia (all *P >* 0.055) (Table 2 and Figure 3). However, reductions in phase were noted across the cardiac cycle when comparing hypercapnic to eucapnic stages (all *P <* 0.009, all LR* P <* 0.001), except for diastolic PCA and systolic MCA during 0.10 Hz SSMs (*P *= 0.132 and *P *= 0.055, respectively) (Table 2 and Figure 4). Gain measures showed differences in diastolic MCA (*P *= 0.039, LR *P <* 0.001) and mean MCA (*P <* 0.001, LR* P <* 0.001) at 0.05 Hz (Table 2 and Figure 5). Notably, reductions in nGain were observed during hypercapnic 0.10 Hz SSMs within diastole MCA (*P *= 0.013, LR *P < *0.001), diastole PCA (*P <* 0.001, LR* P <* 0.001), mean MCA (*P <* 0.001, LR* P <* 0.001) and mean PCA (*P *= 0.002, LR *<* 0.001) (Table 2 and Figure 6). Moreover, reductions in nGain were evident during hypercapnic 0.05 Hz SSMs within diastole MCA (*P *= 0.039, LR* P *= 0.001), diastole PCA (*P *= 0.031, LR* P <* 0.001) and mean PCA (*P *= 0.023, LR* P *= 0.002) (Table 2 and Figure 6).

When comparing eucapnic to hypocapnic stages, differences in PSD were observed in both mean MCA (*P <* 0.001, LR* P <* 0.001) and PCA (*P *= 0.001, LR* P *= 0.012) during 0.05 Hz SSMs, while no differences in PSD across the cardiac cycle were noted during the 0.10 Hz SSMs (all *P >* 0.059) (Table 2). During hypocapnic stages, differences in coherence were observed within systolic PCA measures during 0.10 Hz SSMs (*P *= 0.018*, LR P *= 0.028), with all other *P*‐values greater than 0.146 (Table 2 and Figure 3). Additionally, the phase was higher across the cardiac cycle during hypocapnic stages (all *P <* 0.037, all *LR P <* 0.001), except for diastolic PCA measures during 0.10 Hz SSMs (*P *= 0.337) (Table 2 and Figure 4). Moreover, decreases in absolute gain were noted during hypocapnic 0.05 Hz within diastolic MCA (*P *= 0.001, LR* P <* 0.001), mean MCA (*P <* 0.001, LR *P <* 0.001) and mean PCA (*P *= 0.003, LR* P <* 0.001), while decreases in absolute gain were only evident in mean MCA (*P *= 0.41, LR* P *= 0.034) during 0.10 Hz SSMs (Table 2 and Figure 5). Finally, comparing nGain during the hypocapnic stage, only diastolic PCA was changed during 0.05 Hz SSM (*P *= 0.001, LR* P <* 0.001). However, during hypocapnic 0.10 Hz SSMs, nGain was higher across the cardiac cycle (all *P <* 0.007, all LR* P <* 0.001), except for systolic PCA (*P *= 0.997) (Table 2 and Figure 6).

### 3.2 Biological sex differences

Biological sex differences were observed across conditions during 0.05 Hz SSM within the systolic BP PSD metrics (*P *= 0.48, LR* P *= 0.068) (Table 2). In contrast, no other biological sex discrepancies were noted within PSD metrics across conditions and the cardiac cycle (all *P >* 0.149) (Table 2). No biological sex differences were found within coherence metrics across all conditions and across the cardiac cycle (all *P >* 0.129) (Table 2 and Figure 3). Furthermore, no biological sex differences in phase metrics were observed across both SSM frequencies and all conditions (all *P >* 0.160) (Table 2 and Figure 4). Gain metrics during 0.05 Hz SSMs showed no difference between sexes (all *P >* 0.205); however, biological sex differences in gain were observed during 0.10 Hz SSMs within systole MCA (*P *= 0.007, LR* P *= 0.032) and PCA (*P *= 0.016, LR* P *= 0.015) measures (Table 2 and Figure 5). Moreover, biological sex differences were apparent in diastole MCA (*P *= 0.025, LR* P *= 0.001) and PCA (*P *= 0.023, LR* P <* 0.001) in nGain metrics across 0.05 Hz SSMs (Table 2 and Figure 6). Finally, biological sex differences were noted during 0.10 Hz SSMs within systolic MCA measures (*P *= 0.041, LR* P *= 0.001) (Table 2 and Figure 6).

## 4 DISCUSSION

This study aimed to investigate the influence of CVR on dCA during repeated SSMs and to explore potential sex differences in dCA responses across eucapnic, hypercapnic and hypocapnic trials throughout the cardiac cycle. The analysis compared hypercapnic and hypocapnic stages to eucapnic trials (serving as the reference) and compared male to female participants (also serving as the reference) across all stages. The key findings were (1) phase was impacted by hypercapnia (substantial decreases) and hypocapnia (drastic increases), and (2) consistent with the literature on SSMs at 0.10 Hz (Newel et al., 2022) in eucapnic conditions, biological sex differences were observed across all 0.10 Hz conditions within the systolic aspect of the cardiac cycle, as MCA systolic gain and nGain were lower in males compared to females. These findings offer potential insights into known biological sex differences in orthostatic intolerance (Fedorowski, 2019) and ischaemic stroke, diseases that are already linked to dCA impairment (Xiong et al., 2017).

### 4.1 Comparisons with previous literature

The current investigation builds upon the first study in this domain by Birch et al. (1995), which examined repeated SSMs during eucapnic, hypercapnic and hypocapnic conditions. Similar to the current study, reductions in phase metrics during hypercapnia and drastic increases in phase during hypocapnia were observed (Figure 4). Moreover, a previous case study by Barnes et al. (2021) investigated the impact of hypercapnia during repeated SSMs in a single participant. Interestingly, the hypercapnic stage compared to the poikilocapnic stage demonstrated a reduction in phase (Barnes et al., 2021). One possible physiological explanation for the decreased phase is hypercapnia induces vasodilatation, limiting the ability of cerebral blood vessels to further dilate in response to changes in blood pressure (Harper & Glass, 1965; Iwabuchi et al., 1973). This physiological phenomenon has been termed ‘vasodilatory reserve’ (Liu et al., 2019). Similarly, the current investigation observed a large reduction in MCA and PCA phase metrics during 0.05 and 0.10 Hz SSMs in both females and males (Figure 4).

In a prior study by Smirl, Tzeng et al. (2014), participants performed a similar repeated SSM protocol under eucapnic, indomethacin, and hypocapnic conditions. During hypocapnic SSM trials with frequencies matching the current investigation (0.05 and 0.10 Hz), MCA and PCA velocities dropped (∼20–30%), independent of blood pressure changes (Smirl, Tzeng et al., 2014). Further, increases in cerebrovascular reactivity index were associated with elevated phase and reduced absolute gain for the mean aspect of the cardiac cycle (Smirl, Tzeng et al., 2014). Consistent with the findings by Smirl, Tzeng et al. (2014), the current investigation also noted an increase in TFA phase metrics within both the MCA and PCA in the mean aspect of the cardiac cycle and built upon these findings by also showing there were no changes noted for the diastolic PCA phase metrics (Figure 4). However, the results from the current investigation differ from those of Smirl, Tzeng et al. (2014), as reductions in gain metrics were solely observed in diastolic MCA, mean MCA and mean PCA during 0.05 Hz SSMs, and mean MCA during 0.10 Hz SSMs (Figure 5). Furthermore, the current investigation demonstrated differences during hypocapnic SSMs for all 0.10 Hz SSMs nGain metrics except systolic PCA values (Figure 6), which differs from the study conducted by Smirl, Tzeng, and colleagues (2014) who reported no differences between nGain metrics during hypocapnic 0.10 Hz SSMs.

Several studies have examined TFA metrics and biological sex disparities under eucapnic conditions. Labrecque et al. (2019) and Newel et al. (2022) found females typically demonstrate higher dCA gain values compared to males and a lower dCA phase in the PCA during repeated SSMs at 0.10 Hz. Additionally, it has been observed that most biological sex differences manifest during the systolic component of the cardiac cycle, particularly during 0.10 Hz SSMs (Favre & Serrador, 2019; Labrecque et al., 2019; Newel et al., 2022). The current investigation aligns with these findings and further demonstrates there are biological sex differences across hypercapnic, hypocapnic as well as eucapnic conditions in both absolute MCA and PCA gain, as well as MCA nGain metrics (Figures 5 and 6).

### 4.2 Physiological underpinnings of the tasks

The cerebrovasculature is highly sensitive to changes in blood CO_2_ levels, as shown in previous research (Ainslie et al., 2005; Ito et al., 2003; Kety & Schmidt, 1948). Reductions in $P_{\mathrm{aC}O_{2}}$ (hypocapnia) lead to cerebral vasoconstriction and a decrease in CBF (Kety & Schmidt, 1948). This vasoconstriction is due to changes in intra‐ and extracellular pH caused by hypocapnia (Jensen et al., 1988; Lambertsen et al., 1961). Additionally, hypocapnia may enhance cerebral autoregulation by widening the cerebral autoregulation curve and causing a rightward shift, preventing cerebral hyperperfusion (Ainslie et al., 2007). The rightward shift may explain the enhanced dCA timing response observed in the current (Figure 4) and previous studies (Aaslid et al., 1989; Smirl, Tzeng et al., 2014). However, it should be noted the entrenched dogma regarding Lassen's curve (Lassen, 1959) for cerebral autoregulation has been challenged with researchers proposing there is a more limited plateau (or absent) region than previously stated (reviewed in: Brassard et al., 2021).

Conversely, hypercapnia triggers cerebrovascular vasodilatation due to increased production of nitric oxide, a potent vasodilator, by endothelial cells in response to elevated arterial CO_2_ levels (Ito et al., 2003; Kety & Schmidt, 1948; Toda et al., 2009). The reduction in arterial tone may explain the decreases in phase during SSMs, indicating a delayed dCA response, as seen in the current (Figure 4) and previous research (Barnes et al., 2021; Carter et al., 2020; Panerai et al., 1999).

Across conditions, biological sex differences were noted in systolic MCA and PCA gain and MCA nGain metrics, with females displaying higher gain and nGain throughout the 0.10 Hz SSMs (Table 2, Figures 5 and 6). One possible explanation for the differences in systolic coherence and gain metrics is the hormonal influence on cerebrovascular function (Favre & Serrador, 2019; Krause et al., 2006; Skinner et al., 2021). Previous studies have indicated that oestrogen exerts vasodilatory effects on cerebral blood vessels (Favre & Serrador, 2019) and may improve endothelial function (Krause et al., 2006). However, this may depend on the cycle phase, as the autoregulatory index appears lower during high hormone phases than during low hormone phases (Favre & Serrador, 2019; Skinner et al., 2021). Furthermore, during 0.10 Hz SSMs, the frequency is too rapid for the baroreflex to mediate changes in the cerebral pressure–flow relationship (Newel et al., 2022). This, combined with the hormonal influences, may explain the systolic biological sex differences observed in the current investigation. Finally, biological sex differences were noted with 0.05 Hz MCA and PCA nGain metrics within the diastolic component of the cardiac cycle, with males showing greater nGain than females across the three conditions (Table 2 and Figure 6). Previous studies have indicated that testosterone may increase baroreflex sensitivity during transient hypertension compared to females (Fu & Ogoh, 2019), which may explain the enhanced gain observed in diastolic nGain metrics. As such, future research is warranted to observe and confirm how the various phases of the menstrual cycle and/or contraceptive use strategies impact dCA during repeated SSM across the cardiac cycle.

### 4.3 Future directions

Previous studies have suggested that increased gain metrics during repeated SSMs are associated with attenuated dCA abilities (Favre & Serrador, 2019; Labrecque et al., 2019; Newel et al., 2022). However, it has also been proposed that the increased resting CBF observed in females may mitigate the effects of attenuated dCA responses, allowing females to effectively respond to rapid changes in blood pressure induced by repeated SSMs (Labrecque et al., 2019). Subsequently, it is suggested females may be more resilient and able to better accommodate fluctuations in systemic blood pressure. Based on the findings from the current investigation, it is plausible that this enhanced resilience to changes in blood pressure may lower susceptibility conditions associated with impaired cerebral perfusion, including transient ischaemic attacks and ischaemic strokes. Additionally, research supports this notion, as the incidence of stroke is higher in young males compared to females (Abdu & Seyoum, 2022). Furthermore, there is a growing focus in the research field to understand how female contraceptive use may also influence the changes noted in the current research findings (Barranca et al., 2023; Johnson et al., 2023; Pereira et al., 2022; Skinner et al., 2021). However, future work is warranted to confirm such propositions.

### 4.4 Limitations

The primary limitation of this study is the use of TCD to insonate the participants’ MCA and PCA throughout the protocol, which provides a measure of CBv and does not directly quantify CBF. TCD lacks the capability to directly quantify vessel diameter, and relies on the assumption vessel diameter remains constant to be effectively used as a surrogate for CBF (Ainslie & Hoiland, 2014). Previous studies employing functional magnetic resonance imaging have observed that CBv remains relatively constant within ∼8 mmHg of eucapnic values (Coverdale et al., 2014; Verbree et al., 2014). Despite this, during our investigation, $P_{\mathrm{ETC}O_{2}}$ was deliberately clamped at 40 mmHg for eucapnia, 55 mmHg for hypercapnia and 25 mmHg for hypocapnia, which exceeds the ∼8 mmHg recommendation. This indicates the current CBv measures may provide good indexes of CBF for eucapnic data, underestimate the CBF changes by ∼20–25% for the included hypercapnic data, and overestimate the CBF changes by ∼10% for hypocapnic data (Ainslie & Hoiland, 2014). Therefore, despite the potential for underestimation or overestimation, the true CBF effects are likely more pronounced than reported. Nonetheless, the observed results still align with trends observed in previous studies, lending confidence to the current findings (Aaslid et al., 1989; Barnes et al., 2021; Maggio et al., 2013).

Additionally, the study primarily included healthy, young adults, which may limit the generalizability of the results to younger, older or clinical populations. Moreover, the potential impact of factors such as the various aspects of the menstrual cycle phase or cardiorespiratory fitness status on the outcome metrics remains unclear. However, recent research has indicated minimal influence of the menstrual cycle on dCA (Favre & Serrador, 2019; Johnson et al., 2023; Korad et al., 2022), thereby bolstering confidence the menstrual cycle and/or contraceptive use would likely have a limited impact on the current findings. Furthermore, each participant completes all three stages of the protocol during the same visit, effectively serving as their own eucapnic controls for the hypercapnic and hypocapnic trials, helping to mitigate the impact of any confounding factors within the task comparisons (Charness et al., 2012).

### 4.5 Conclusion

The present study aimed to investigate the interaction between CVR and dCA responses, particularly during repeated SSMs performed at 0.05 and 0.10 Hz, while also examining potential differences between biological sexes. Collectively, the findings confirm the substantial effects altered CO_2_ tension have on the cerebrovasculature and demonstrate how they will impact another major regulatory control mechanisms for CBF, dCA. Specifically, the results indicated delayed dCA responses to hypercapnic stimuli and enhanced dCA during hypocapnic trials, characterized by large decreases and increases in phase, respectively. Therefore, it is imperative for physiological investigations into dCA to closely monitor and minimize variations in CO_2_ tensions as these will have profound impacts on the outcome metrics.

Furthermore, the findings also revealed there are biological sex‐based disparities in dCA which appear most by sympathetic modulation as 0.10 Hz SSM difference metrics during 0.10 Hz SSM were observed in systolic gain and nGain TFA metrics across the middle and posterior cerebral arterial beds. Furthermore, these differences were evident under both hypercapnic and hypocapnic conditions, indicating they were not artifacts from the altered CO_2_ tensions. Future studies are warranted to discern whether these biological sex differences in TFA metrics may underpin differences in incidence rates of certain clinical conditions and the potential influences of contraceptive use on these dCA metrics.

## AUTHOR CONTRIBUTIONS

Nathan E. Johnson, Joel S. Burma, and Jonathan D. Smirl conceptualized and designed the study. Nathan E. Johnson, Joel S. Burma, Elizabeth K.S. Fletcher, and Joshua J. Burkart performed the experiments. Nathan E. Johnson and Joel S. Burma conducted the data analysis. Nathan E. Johnson and Jonathan D. Smirl wrote the manuscript. Joel S. Burma, Matthew G. Neill, Joshua J. Burkart, and Elizabeth K.S. Fletcher contributed to editing and revising the manuscript. All authors have approved the final version of the manuscript and agree to be accountable for all aspects of the work, ensuring that any questions related to the accuracy or integrity of any part of the work are appropriately investigated and resolved. All contributing and corresponding authors qualify for authorship, and all those who qualify for authorship are listed.

## CONFLICT OF INTEREST

None declared.

## Data Availability

The data supporting the findings from this manuscript are available upon reasonable request to the corresponding author (M.G.N.).
